# A genome wide SNP genotyping study in the Tunisian population: specific reporting on a subset of common breast cancer risk loci

**DOI:** 10.1186/s12885-018-5133-8

**Published:** 2018-12-29

**Authors:** Yosr Hamdi, Mariem Ben Rekaya, Shan Jingxuan, Majdi Nagara, Olfa Messaoud, Amel Benammar Elgaaied, Ridha Mrad, Lotfi Chouchane, Mohamed Samir Boubaker, Sonia Abdelhak, Hamouda Boussen, Lilia Romdhane

**Affiliations:** 1 Laboratory of biomedical genomics and oncogenetics, Institut Pasteur de Tunis, Université Tunis El Manar, 13, Place Pasteur BP 74, 1002 Tunis, Belvédère Tunisie; 2 Department of Genetic Medicine, Weill Cornell Medical College-Qatar, Doha, Qatar; 3 Laboratory of Genetics, Immunology and Human Pathology, Department of Biology, Faculty of Sciences of Tunis, University of Tunis El Manar, Tunis, Tunisia; 4 Department of Human Genetics, Charles Nicolle Hospital, Tunis, Tunisia; 5 Medical Oncology Department, Abderrahmen Mami Hospital, Ariana, Tunisia; 6 Department of Biology, Faculty of Science of Bizerte, Université Tunis Carthage, Tunis, Tunisia

**Keywords:** Breast cancer susceptibility, Haplotype analysis, Population genetics, Functional analysis

## Abstract

**Background:**

Breast cancer is the most common cancer in women worldwide. Around 50% of breast cancer familial risk has been so far explained by known susceptibility alleles with variable levels of risk and prevalence. The vast majority of these breast cancer associated variations reported to date are from populations of European ancestry. In spite of its heterogeneity and genetic wealth, North-African populations have not been studied by the HapMap and the 1000Genomes projects. Thus, very little is known about the genetic architecture of these populations.

**Methods:**

This study aimed to investigate a subset of common breast cancer loci in the general Tunisian population and to compare their genetic composition to those of other ethnic groups. We undertook a genome-wide haplotype study by genotyping 135 Tunisian subjects using the Affymetrix 6.0-Array. We compared Tunisian allele frequencies and linkage disequilibrium patterns to those of HapMap populations and we performed a comprehensive assessment of the functional effects of several selected variants.

**Results:**

Haplotype analyses showed that at risk haplotypes on 2p24, 4q21, 6q25, 9q31, 10q26, 11p15, 11q13 and 14q32 loci are considerably frequent in the Tunisian population (> 20%). Allele frequency comparison showed that the frequency of rs13329835 is significantly different between Tunisian and all other HapMap populations. LD-blocks and Principle Component Analysis revealed that the genetic characteristics of breast cancer variants in the Tunisian, and so probably the North-African populations, are more similar to those of Europeans than Africans.

Using eQTl analysis, we characterized rs9911630 as the most strongly expression-associated SNP that seems to affect the expression levels of *BRCA1* and two long non coding RNAs (*NBR2* and *LINC008854*). Additional *in-silico* analysis also suggested a potential functional significance of this variant.

**Conclusions:**

We illustrated the utility of combining haplotype analysis in diverse ethnic groups with functional analysis to explore breast cancer genetic architecture in Tunisia. Results presented in this study provide the first report on a large number of common breast cancer genetic polymorphisms in the Tunisian population which may establish a baseline database to guide future association studies in North Africa.

**Electronic supplementary material:**

The online version of this article (10.1186/s12885-018-5133-8) contains supplementary material, which is available to authorized users.

## Background

Breast cancer is a complex disease with a strong heritable component. Numerous genetic, hormonal and external environmental factors are involved in breast cancer etiology [1].

Currently, half of the inherited susceptibility to breast cancer is explained by a combination of high, intermediate, and low-risk alleles [2]. Rare high risk alleles such as *BRCA1* [3, 4], *BRCA2* [5, 6], *TP53* [7], *STK11, PTEN* and *CDH1* explain approximately 20–30% of the inherited susceptibility, intermediate-risk alleles in genes such as *CHEK2, ATM, BRIP1(FANCJ)* [8–10] and *PALB2 (FANCN)* [11–15] explain an additional 5%, while common lower penetrance alleles explain approximately 16% of the breast cancer risk [16–23]. Thus, many additional loci remain to be identified [24–26].

In North Africa, breast cancer is the most common cancer among women, representing 25 to 35% of all female cancers [27]. Compared to Western countries, the incidence of breast cancer in North-African countries (Tunisia, Algeria, Morocco, Libya and Egypt) is lower [28, 29]. However, it is reported that breast cancer is more aggressive in North Africa than in Western countries with notably large proportions of young patients [30–32].

These large differences are mainly explained by different genetic architectures between populations. Initiatives such as the international HapMap and the 1000 Genomes projects [33, 34] provided an unprecedented opportunity to systematically analyze genetic differences and similarities between human populations from European, Asian, and Sub-Saharan origins. It is well known that any given haplotype frequency may differ from one population to another because of different Linkage Disequilibrium (LD) structures and variable genetic variant frequencies among populations [33]. Analysing differences in genetic loci patterns between ethnicities may help to decode the biological mechanisms by which the risk associated variant at a susceptibility locus causes breast cancer [34–37]. Indeed, the high level of haplotype diversity in African populations may facilitate fine-mapping of causal variants that underlie disease associations [38–40]. For instance, fine mapping in the African-American population contributed to the localization of a causal variant in *FGFR2*, a low penetrance breast cancer susceptibility locus [41]. Therefore, studying other African populations may bring new insights on breast cancer genetic knowledge.

The Tunisian population (TUN), is a heterogeneous and admixed population from African and European origins [42]. The particular structure of the Tunisian population is also due to a relatively high rate of consanguinity that has an impact on the incidence of monogenic diseases such as those predisposing to cancer [43]. Despite its genetic wealth, few breast cancer genetic studies have been performed in the TUN population such as those that focused on the identification of the mutational spectrum of *BRCA1* and *BRCA2* [44, 45]. Thus, little is known about the involvement of other genes of high and moderate penetrance and much less for common variants regarding their frequencies, correlation coefficients and association with breast cancer risk in Tunisia. Indeed, a study investigating breast cancer risk in Tunisia associated with 9 risk SNPs has been performed [46]. Because of the limited number of breast cancer risk SNPs that have been investigated so far in the Tunisian population, this current study aim to extend the number of common genetic variants investigated in the general healthy Tunisian population and to compare their characteristics between different ethnic groups.

## Methods

### Study population and DNA extraction

A total of 165 healthy unrelated Tunisian individuals originating from different regions of Tunisia (North, Center and South) have been recruited from the department of Radiation Oncology of Sousse Hospital (Sousse, Tunisia) and from Pasteur Institute (Tunis, Tunisia). Males (45%) and females (55%) (Reflecting the sex ratio of the general Tunisian population) having no evidence of any personal or familial history of breast cancer or any other known malignancies have been included. Participants had a mean age of 48 ± 10 years. All individuals signed informed consents. Ethical approval according to the Declaration of Helsinki Principles was obtained from the biomedical ethics committee of Pasteur Institute of Tunis (PV09/06, IRB# 0000000044).

Genomic DNA was extracted from peripheral blood leukocytes by a “Salting-out” procedure [47]. 5 ml of blood was mixed with Triton lysis buffer. Leukocytes were spun down and washed with H2O.The pellet was incubated with proteinase K and subsequently salted out using a saturated NaCl solution. Precipitated proteins were removed by centrifugation. The DNA pellet was dissolved in 400 μl of sterile distilled water. DNA concentration and quality were analyzed by the Nanodrop 2000.

### Genotyping and quality control steps

The initial individual subset was 165 healthy Tunisians. We first performed a sorting step based on the DNA quality and concentration. At this step we excluded 30 individuals due to their bad DNA quality and thus a total of 135 samples have been successfully genotyped. Standard quality control measures were applied across all genotyped samples. For quality control purposes,~ 2% of samples were duplicated.

Genome-wide scanning was applied with Affymetrix Genome-Wide Human SNP Array 6.0 following the manufacturer’s protocol. After genotype calling using the R package CRLMM [48], the total SNPs on Affymetrix array were subjected to quality control. 291,195 SNPs have been excluded because of low chip design scores and low call frequency (< 95%). In fact, the Affymetrix array 6.0 has been designed based on HapMap data providing a lower level of genome coverage in population from African ancestry than European and Asian populations. 80.624 SNPs have been excluded because of low minor allele frequency (< 5%) and evidence of deviation from Hardy Weinberg Equilibrium. The final SNP set included 534,781 SNPs for genome-wide analysis.

In addition, genotype data of the selected breast cancer polymorphisms were also extracted for populations from the HapMap3 project including Europeans (Utah residents with European ancestry (CEU) and Toscans in Italy (TSI)), Asians (Han Chinese in Beijing (CHB), Chinese in Metropolitan Denver (CHD), Gujarati Indians in Houston (GIH) and Japanese in Tokyo (JPT)), Africans (Yoruba in Nigeria (YRI), Maasai in Kinyawa (MKK), Luhya in Kenya (LWK) and African ancestry in Southwest USA (ASW)) as well as Mexicans living in Los Angeles (MEX).

Minor allele frequency (MAF) and departure from Hardy Weinberg equilibrium (HWE) of markers at breast cancer loci were estimated using the SNPassoc R package [49].

### Genotyping data analysis

To assess population stratification, a multidimensional scaling analysis (MDS) was performed as implemented in PLINK 1.07 on the identity-by-state (IBS) matrix of the samples. PLINK was used to carry out the genome-wide analysis of the autosomal SNPs and to perform permutation test to examine the stability of *p*-values. Genotype distributions and pairwise comparisons were evaluated by the chi-square test using a p-value threshold of 0.05.

### LD analysis

Genotype and marker data files were loaded into the Haploview software (http://www.broad.mit.edu/mpg/haploview/) [50]. The r^2^ statistics of the Haploview4.0 software were used for haplotype block identification by calculating the pairwise LD for each sequence variant pair for the 135 genotyped subjects as well as those from the HapMap project. The Gabriel et al. algorithm of block definition was selected [51]. The ‘Tagger’ program from the same software was used to select a minimal set of tagSNPs as if all alleles are captured (frequency > 5%) and was correlated at an r^2^ greater than a 0.8 threshold.

### Haplotype estimation

Haplotype phasing and frequency estimations were performed using Phase 2.1.1 software [52]. This program estimates haplotype frequencies with a Bayesian-based algorithm. Haplotypes were phased using SNPs with MAF ≥5%.

### In silico assessment of functional effects

To predict potential functional impact of selected SNPs, we used web-based algorithms with default settings: Align-GVGD (http://agvgd.iarc.fr/) [53] and SIFT (http://sift.bii.a-star.edu.sg/) mainly based on phylogenetic information and biochemical differences between the reference and variant amino acid for assessing the functional effects of missense variants [54] and the RegulomeDB database (www.regulomedb.org) to evaluate the functional impact of regulatory variants [55]. Publicly available genomic data was also used to annotate variants that showed high RegulomeDB scores. The following regulatory features were obtained for different cell types including breast cancer cells from ENCODE and NIH Roadmap Epigenomics data through the UCSC Genome Browser: Transcription factor ChiP-seq data, altered motifs, eQTL, histone modifications and Chromatin Hidden Markov Modelling (ChromHMM) states.

In order to identify variants that alter micro RNA binding sites, we used the latest miRBase release (v20, June 2013) (http://www.mirbase.org/), the primary microRNA sequence repository that contains 24,521 microRNA loci from 206 species.

### eQTL analysis

rs9911630-*BRCA1* was tested for correlation with nearby gene expression using the eQTL database GENe Expression VARiation (Genevar) from Sanger institute platform [56]. Genotypes and expression data within this database are derived from 3 cell types (fibroblast, lymphoblastoid cell line and T cell) and 3 tissue types (166 adipose, 156 lymphoblastoid cell line and 160 skin) from healthy female twins [56]. eQTL data is available for 8 different populations from European, Asian and African origins. Differences in the distribution of normalized expression levels between genotypes were compared using a linear regression model. To avoid false positive associations due to multiple tests, we set a significance threshold of *p* < 10^− 3^ and assessed significance using 10,000-folds permutation.

We also used the Genotype-Tissue Expression (GTEx) database (http://gtexportal.org) in order to assess the correlation between selected breast cancer associated variants and gene expression levels. GTEx provides data on the relationship between the global RNA expression within multiple human tissues and variants genotypes. Variations in gene expression that are highly correlated with genetic variation can be identified as eQTLs. Because eQTLs are known to be tissue specific, we assessed the correlation between breast cancer associated variants and gene expression levels in breast mammary tissues from the GTEx database.

### Principle components analysis

Minor allele frequencies of the selected SNPs in the different studied populations were used for principal components analysis (PCA) in order to study the different breast cancer genetic structures. Principal components analysis aims to synthesize information contained in a set of *n* observed variables (M_1_, …, M_n_) by seeking a new set of k (k < n) uncorrelated variables. The new variables, called principal components, are a linear combination of the observed variables. PCAs have been dressed with respect to the following criteria: percentage of missing genotypes, MAF > 0.05, HWE *p*-value > 0.0001 and r^2^ < 0.6. The resulting matrix was used for PCA calculation using the dudi.pca function from R packages. The plotting of the two first PCA were performed using the Factoextra R packages.

## Results

In the present study, we investigated the breast cancer genetic architecture in a set of 135 subjects from the general Tunisian population by analyzing several previously identified breast cancer associated variations. Additional file 1: Table S1. summarizes the 90 breast cancer susceptibility loci that have been investigated in this current study. The studied genomic regions have been selected as follow: for the high and moderate penetrance genes we selected a genomic region that covers the LD block containing each gene in order to perform haplotype analysis. For low penetrance loci (SNPs) we studied a genomic region that covers 20 kb upstream and downstream of each SNP. We selected 20 kb on both sides in order to avoid overlap between variants that are located on the same locus. Thus, a 7 Mb genomic region have been spanned for each of the 135 studied subjects.

One thousand five hundred eighty-seven variants directly genotyped on the array and located on these 90 selected loci have been included in this study. Among them, 28 SNPs are known to be associated with overall breast cancer risk (Table 1) including 3 SNPs already identified to be associated with breast cancer risk in the Tunisian population namely rs1219648, rs2981582 in *FGFR2* and rs8051542 in *TOX3* [46].

**Table 1:** Distribution, minor allele frequencies and functional prediction of the selected breast cancer common variants

Locus	Marker ID	Alleles	GMA	GMAF	TUN (freq)	Location	Score	eQTL associations	Predicted function
1q32.1	rs4245739	A/C	C	0.2141	0,333	Intergenic	6	No association	3UTR MDM4 miR-191 target site and results in decreased MDM4 expression
2p24.1	rs12710696	T/C	T	0.4455	51,1	Intergenic	4	No association	TF binding and DNase peak
2q31.1	rs1550623	A/G	G	0.1711	0,24	Intergenic	4	No association	TF binding and DNase peak
4q21	rs1494961	C/T	C	0.3355	52,6	Exonic *HELQ* c.916G > A	1f	No association	Tolerated
4q21	rs11099601	C/T	C	0.33613	53,4	3 UTR *FAM175A* c.413 C > T	1f	*p* = 1.94 × 10^−21^ with *MRPS18C*	DAE eQTL and TF binding / DNase peak
4q34.1	rs6828523	C/A	A	0.2468	0,179	Intronic, *ADAM29* c.-450-5711C > A	No data	No association	No data
6p23	rs204247	A/G	G	0.4321	0,37	Intergenic (11 kb 5′-*RANBP9*)	6	No association	Minimal functional evidence
6q25.1	rs2046210	G/A	A	0.4121	0,45	Intergenic, 6 kb 3’-*CCDC170*	6	No association	Minimal functional evidence
7q35	rs720475	G/A	A	0.1478	0,263	Intronic; *ARHGEF5* c.4531 + 646G > A	5	*p* = 1.4 × 10^−6^ with *ARHGEF34P* gene and *p* = 4.2 10^− 6^ for *OR2A9P* gene	TF binding or DNase peak
9q31.2	rs10759243	C/A	A	0.4607	0,477	Intergenic (53 kb 5′-*KLF4*)	No data	No association	No data
10p12.31	rs7072776	G/A	A	0.3055	0,441	Intergenic (382 bp 3′ *MLLT10*)	5	No association	TF binding or DNase peak
10p15.1	rs2380205	C/T	T	0.3750	0,48	Intergenic, 2.6 kb 5′-*GDI2*	3a	No association	TF binding, any motif and DNase peak
10q22.3	rs704010	C/T	T	0.2674	0,322	Intronic; *ZMIZ1* c.-337 + 12,121 T > C	2b	No association	TF binding, any motif, DNase Footprint and DNase peak
**10q26**	**rs1219648**	A/G	G	0.4089	0,466	Intronic; *FGFR2* c.109 + 7033 T > C	No data	No association	No data
**10q26**	**rs2981582**	G/A	A	0.4038	0,463	Intronic, *FGFR2* c.109 + 906 T > C	5	No association	TF binding or DNase peak
11q13.1	rs3903072	G/T	T	0.3165	0,467	Intergenic (7.4 kb 3′-*CFL1*)	4	*p* = 9 × 10^−6^ for *SNX32*, and *p* = 2.9 × 10^− 5^ for *CTSW*	TF binding and DNase peak
11p15.5	rs3817198	T/C	C	0.2155	0,277	3UTR *LSP1* c. 13 + 200 T > C	5	No association	TF binding or DNase peak
12q24.21	rs1292011	A/G	G	0.4211	0,469	Intergenic	4	No association	TF binding and DNase peak
14q24.1	rs2588809	C/T	T	0.1831	0,298	Intronic *RAD51B* c.757–98,173 T > C	No data	No association	No data
14q32.11	rs941764	A/G	G	0.4193	0,468	Intronic *CCDC88C* c.271–15,014 T > C	4	No association	TF binding and DNase peak
16q12.1	rs3803662	G/A	A	0.4403	0,414	Intergenic, 5′ to *TOX3*	5	No association	TF binding or DNase peak
**16q12.1**	**rs8051542**	C/T	T	0.3133	0,396	Intronic *TOX3* c.88–3168 A > G	5	No association	TF binding or DNase peak
16q23.2	rs13329835	A/G	G	0.2957	0,376	Intronic *CDYL2* c.1007 + 3855 T > C	4	No association	TF binding and DNase peak
17q21	rs9911630	A/G	G	0.4972	0,426	3’of *BRCA1* NC_000017.10:g.41188342A > G	1b	*p* = 1.2 × 10^−23^ for *NBR2* and *p* = 1.3 × 10^−6^ for *CTD-3199 J23.6* and *p* = 6.1 × 10^−6^ for *LINC00854*	eQTL, TF binding, any motif, DNase Footprint and DNase peak
17q21	rs799916	T/G	T	0.4976	0,404	Intronic *BRCA1* c.4097-141A > C	6	*p* = 2.1 × 10^−25^ for *NBR2 p* = 8.3 × 10^−7^ *for CTD-3199 J23.6* and *p =* 3.7 × 10^−6^ for *LINC00854*	Minimal functional evidence
18q11.2	rs1436904	T/G	G	0.3568	0,292	Intronic *CHST9* c.202 + 33413A > C	No data	No association	No data
19p13.11	rs4808801	A/G	G	0.4521	0,404	Intronic *ELL* c.744 + 1247 T > C	1f	p = 1.6 × 10^−5^ for *SSBP4*	eQTL, TF binding and/or DNase peak
22q13.1	rs6001930	T/C	C	0.1414	0,109	Intronic *MKL1* c.-59-16944A > G	5	No association	TF binding or DNase peak

-*GMA* Global Minor Allele, *GMAF* Global Minor Allele Frequency, *TUN (freq)* the frequency of the global minor allele in the Tunisian population, Score: from the RegulomeDB database and score significance provided in the predicted function column, *eQTL association* provided by the GTEx database, *p* the p value of the variants’ eQTL association, *TF* Transcription factor

-The highlighted rows indicate polymorphisms that showed the highest RegulomeDB scores, significant eQTL associations and other functional evidence

-rs1494961 is the only exonic variant in this list, we provided its predicted functional significance using the Sift software

-In bold, SNPs previously identified as associated with breast cancer risk in the Tunisian population

We therefore performed haplotype analysis by constructing LD blocks and phasing haplotypes on the 90 loci. We calculated correlation coefficients and allelic frequencies and characterized haplotype tagging SNPs (htSNPs). This constitutes a genetic database for use in further breast cancer association studies in the Tunisian population.

**Fig. 1:**
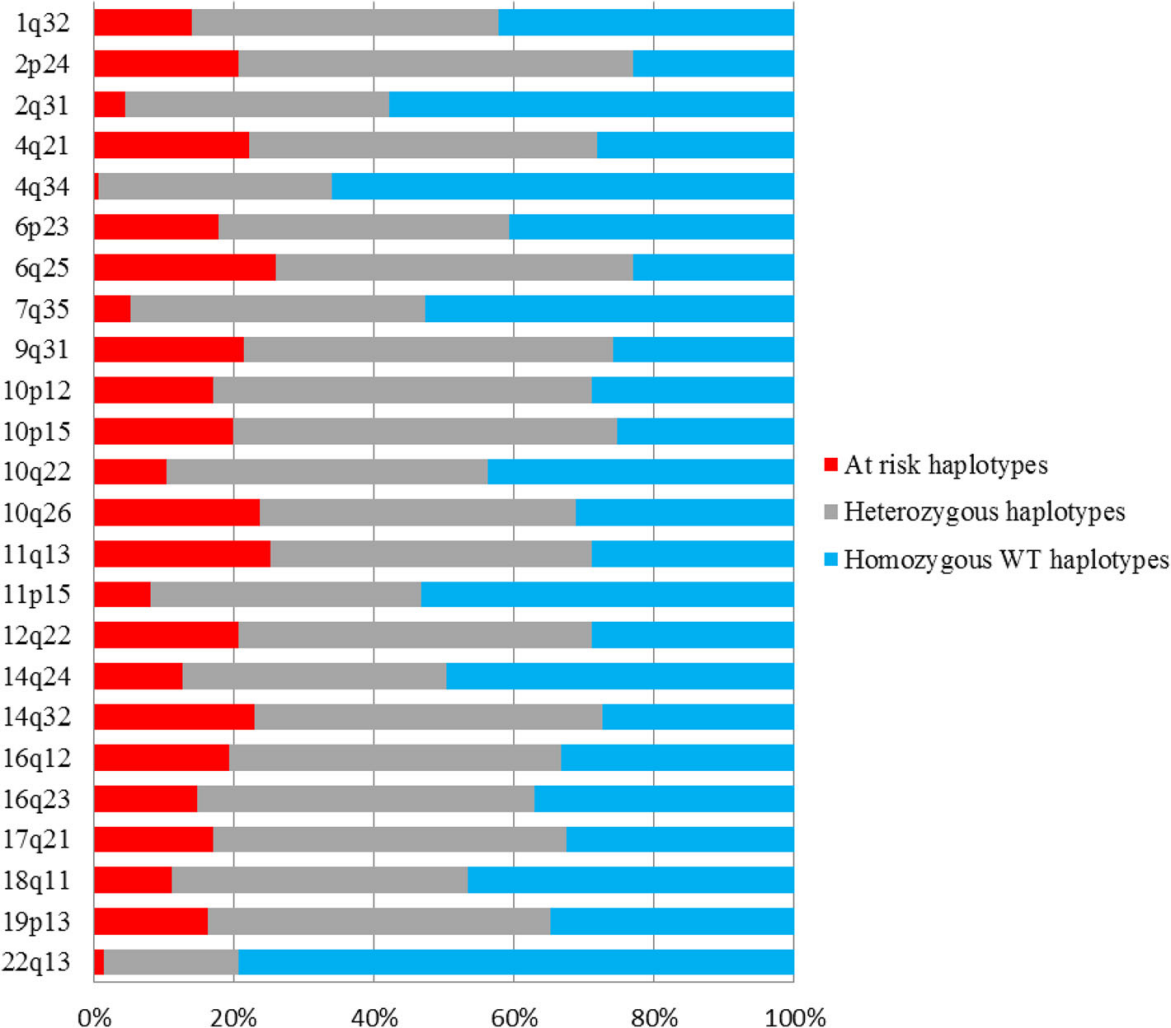
Distribution of breast cancer at risk haplotypes in the general Tunisian population. After phasing the identified haplotypes in the Tunisian population, the frequencies of at risk haplotypes (in red; identified as haplotypes carrying at risk alleles, those that have been identified as associated with the disease and usually considered as the minor alleles) have been calculated. At risk haplotypes with frequency ≥ 5% are considered as relatively common haplotypes

We determined the frequencies, in the Tunisian population, of at risk haplotype that carry at risk alleles of the 28 variants known to be associated with breast cancer risk (Fig. 1). Our results suggest that at risk haplotypes on 2p24, 4q21, 6q25, 9q31, 10q26,11p15, 11q13 and 14q32 loci are considerably frequent in the Tunisian population (haplotype frequency > 20%), however, at risk haplotypes of the 2q31; 4q34, 7q35 and 22q13 loci seem to be rare (frequency < 5%).

Moreover, we explored the functional role of the 28 selected variants. We performed analysis using RegulomeDB to identify DNA features and regulatory elements overlapping this set of variants and by applying a heuristic RegulomeDB score to prioritize candidate functional variants prior to further investigation (for a description of the RegulomeDB scoring scheme and referenced datatypes refer to http://www.regulomedb.org). High RegulomeDB scores were attributed to four SNPs: rs11099601, rs1494961, rs9911630 and rs4808801 (Table 1). Further functional annotations for each of these SNPs are shown in Additional file 1: Table S2.

The highly ranked score “1b” was assigned to the *BRCA1*-rs9911630 variant. The “1b” RegulomeDB score suggests that this variant is a putative functional SNP that may be associated to eQTL evidences. Then, eQTL associations have been assessed for the 28 variants using data from the GTEx database. In breast mammary tissues, significant eQTL associations have been observed for rs720475, rs3903072, rs9911630, rs799916 and rs4808801 (Table 1). The most strongly expression-associated variants were rs9911630 and rs799916, two strongly correlated variants in the *BRCA1* genomic region. Both variants were associated with expression levels of *NBR2, CTD-3199 J23.6* and *LINC00854* genes with a highly significant eQTL evidence for *NBR2,* a *BRCA1* neighbor gene (*p* = 1.2 × 10^− 23^ and *p* = 2.1 × 10^− 25^ for rs9911630 and rs799916, respectively) (Additional file 2: Figure S1).

**Fig. 2:**
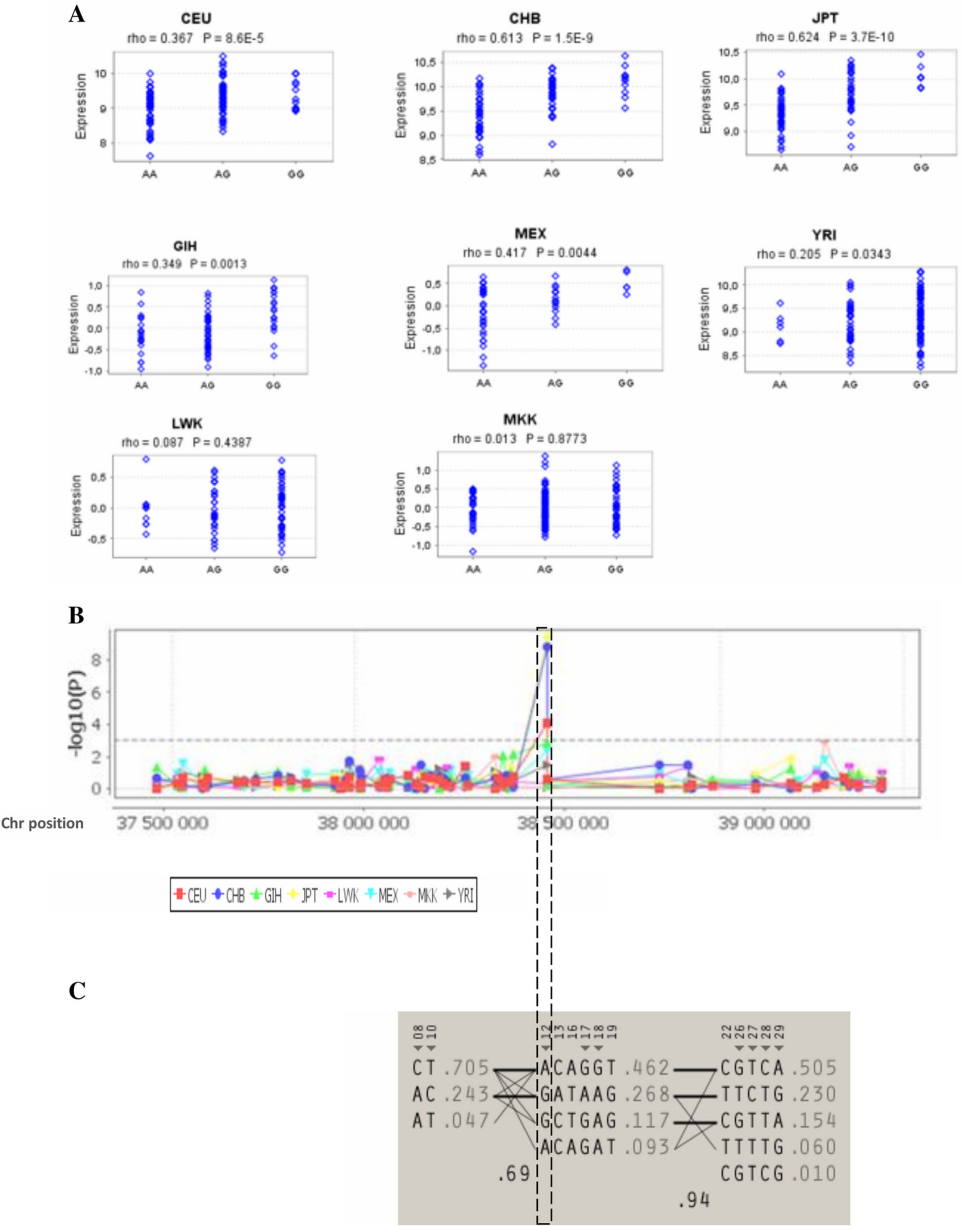
eQTL analysis of the rs9911630- *BRCA1* variant in different populations. **a** rs9911630 was tested for correlation with *BRCA1* gene expression using the eQTL database GENe Expression VARiation (Genevar) from Sanger institute plateform. eQTLs in 8 different HapMap populations are showed in this figure (CEU: Utah residents with Northern and Western European ancestry from the CEPH, CHB: Han Chinese in Beijing, China; JPT: Japanese in Tokyo, Japan; GIH: Gujarati Indians in Houston, MEX: Mexican ancestry in Los Angeles, California; YRI: Yoruba in Ibadan, Nigeria; LWK: Luhya in Webuye, Kenya; MKK: Maasai in Kinyawa, Kenya). The 8 Box plots represent *BRCA1* expression levels on the y axis with respect to the rs9911630 genotypes showed on the x axis. *P* values (P) < 0.05 means significant eQTL associations. **b** eQTL association results across different HapMap populations are shown on the y axis (log10). The chromosomal position of the *BRCA1* gene (chr17:38449840–38,530,994 according to NCBI36/hg18 assembly) is shown on the x axis. eQTL peaks represent the significant eQTL correlation that have been observed for rs9911630 in Caucasian, Chinese and Japanese samples*.* **c** Haplotype blocks constructed with variants identified in the Tunisian subjects showing a frequency ≥ 5%. Tagging SNPs identified on a block-by-block basis are denoted with an arrow below the SNP number. Tunisian haplotype frequencies are displayed on the right of each haplotype combination, while the level of recombination is displayed below the connections between the blocks. Thick connections represent haplotypes with frequencies ≥5%, while frequencies below 5% are represented by thin lines. Variant #12 is rs9911630

Based on its interesting RegulomeDB score and the significant eQTL associations, we undertook further analysis for rs9911630. Using the Genevar platform, we assessed eQTL associations for rs9911630 in 8 populations. Data showed that rs9911630 is significantly associated with the expression level of *BRCA1 gene (ILMN_173827-ENSG00000012048-BRCA1)* in non-African populations (Asian (JPT and CHB) and Caucasian population (CEU)) but not in Africans*.* (Fig. 2a and b). Consequently, we compared the allelic frequencies of rs9911630 between these different populations.

Consistently with eQTL results, the frequency of rs9911630 is significantly different between Africans and non-African populations. Indeed, the “G” minor allele of rs9911630 in European and Asians shifted to a major allele in Africans (Additional file 1: Table S3). The allelic frequency of rs9911630 in Tunisia is significantly different from Africans and Asians and not different from Europeans and haplotype analysis using Haploview tagger tool showed that rs9911630 is a haplotype tagging SNP in the Tunisian population (Fig. 2c).

In silico predictions of micro RNA binding sites of rs9911630, as a *BRCA1*–3′ variant, have been also performed using mirBase. In silico analysis predicts that rs9911630 alters the binding sites of three non human microRNAs by the gain of mse-miR-2766 binding site and the loss of bmo-miR3287 and ssa-miR-19d-5p binding sites (Additional file 2: Figure S2; Additional file 1: Table S4).

Based on the different allelic frequencies of rs9911630 (A > G) in the different ethnic groups investigated in this study, we suggested that the effect of rs9911630 on the microRNAs binding sites will differ from one population to another. Indeed, in European and Asian populations, rs9911630 would cause a gain of miR-2766 binding and a loss of bmo-miR3287 and ssa-miR-19d-5p Micro-RNAs. However, in Africans, rs9911630 would act in a different way by promoting the binding of bmo-miR3287 and ssa-miR-19d-5p and loosing the binding site of miR-2766.

**Fig. 3:**
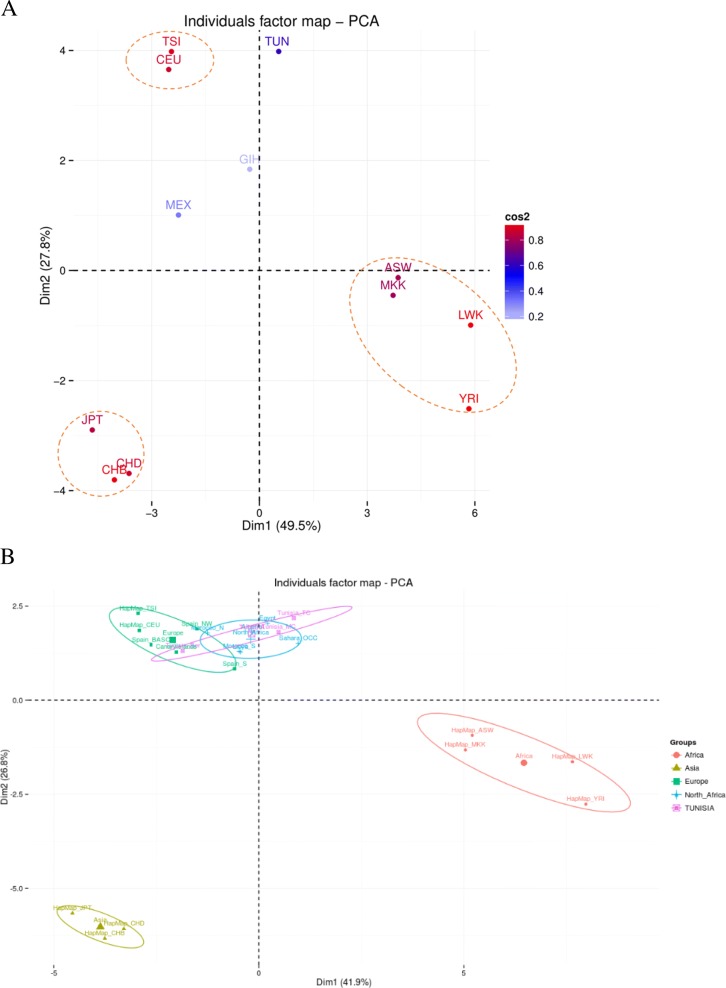
Principle component analysis showing population distribution based on the MAF of the 28 selected variants. **a** Tunisian and HapMap populations. **b** Other sub-populations from North Africa and other ethnic groups

Finally, we used the chi-square statistical test to compare the genetic characteristics of the selected 28 SNPs between TUN and other populations (Additional file 1: Table S3). Interestingly, the frequency of rs13329835 seems to differ significantly between the Tunisian population and all other HapMap populations. In addition, three SNPs (rs2046210, rs941764 and rs3803662) showed significant difference in their frequencies between Tunisian and European (CEU + TSI), Asian (CHB + JPT) and African populations (MKK, LWK and YRI). However, no significant differences have been observed between rs3803662 frequencies in Tunisian and in ASW and MEX populations that are considered as admixed populations. For the remaining 24 SNPs, significant differences either with Africans, Europeans or Asians were observed. In addition, in order to investigate the breast cancer genetic architecture in different ethnic groups, we studied the distribution of the studied population using a principle component analysis based on the frequencies of the 28 genotyped variants. PCA revealed clear distinction of the breast cancer architecture among the three human geographic origins (Europe, Asia and Africa) (Fig. 3). Admixed populations such as MEX and GIH seem to cluster in an intermediate position. However, TUN population seems to cluster close to Europeans populations.

Moreover, the first principal component (Fig. 3a) showed that the largest inter-ethnic variability is found between African and non-African populations (49.5%). A set of 15 variants that contributed significantly to the observed genetic variability between African and non-African populations (Dim1) have been characterized (Additional file 2: Figure S3a). rs4808801 seems to be the variant that contributed more to this inter-ethnic variability. rs9911630 also figure in the list of variants that contribute very significantly to the genetic variability between African and non-African populations. PCA results also showed that the variability between European populations (CEU and TSI) and non-European ones, is about 27.8% and a list of 11 variants have been also identified as the most contributors to the genetic variability between these populations (Dim2) (Additional file 2: Figure S3B).

Consistently with the PCA results, we also showed that YRI, LWK and JPT populations are the most contributors to variability between African and non-Africans (Dim 1), and TUN, MEX and GIH are those who contributed less to this variability (Additional file 2: Figure S4A). This represents an additional argument toward the admixed and intermediate classification of these populations.

However, the Tunisian population altogether with the TSI and CHB populations are the most contributors to diversity between Europeans and non-Europeans, which confirms the close genetic relationship between Tunisians and population from European ancestry (Additional file 2: Figure S4B).

In order to explore the genetic relationship between North African and European populations, we performed another PCA using a new matrix that integrates values from additional populations from North Africa (Tunisian Berbers, Moroccans and Egyptians) and Southern Europe mainly from Spain. This second analysis confirmed the close genetic relationship between North African populations and Europeans (Fig. 3b).

## Discussion

Several differences have been observed in breast cancer epidemiological features between populations [57]. This is mainly due to different demographic, environmental and lifestyle factors, but also result from differences in genetic architecture from one population to another. Indeed, it has been shown that American women from African origins (Afro-Americans) are three times more likely than Caucasian Americans to develop the highly aggressive triple-negative and inflammatory breast cancer forms [58]. Moreover, several studies showed that high rate and long history of consanguinity, commonly observed in developing countries, decrease breast cancer incidence rate by decreasing the frequency of mutations in high penetrant breast cancer genes such as *BRCA1* and *BRCA2* [59, 60]. In Tunisia, mutation prevalence of these two genes is considered lower than in Europe with 19.7 and 7.5% for *BRCA1* and *BRCA2* respectively [61]. Medimegh et al. [62] suggested that in the absence of deleterious mutations on *BRCA1*, wild type alleles of *BRCA1* genetic variants seem to decrease the expression level of the *BRCA1* in 50% of familial breast cancer cases through their interaction with micro-RNAs (miRNAs) that are increasingly recognized as mediators in a variety of biological processes including breast cancer [62].

Because of the lack of large studies on common breast cancer genetic variation in the Tunisian population, we undertook this genome wide study with a focus on 90 breast cancer loci. We investigated differences and similarities between these loci in terms of allelic frequencies in more than 11 different populations, we also investigated linkage disequilibrium, correlation ratios and haplotype structure of breast cancer loci in Tunisia. Moreover, in silico functional assessment of the most relevant SNPs have been performed. This study represent the largest and the most complete study on common breast cancer variants in the Tunisian and North African populations.

Genome wide-PCA performed in this current study (data not shown) showed the admixed and intermediate genetic architecture of the Tunisian population. Although it’s geographic belonging to the African continent, the Tunisian genetic characteristics of breast cancer common variants seems to be closer to Europeans than to Africans. The close genetic relationship between Tunisian population and Europeans may be explained by migratory waves that happened in the Mediterranean region since the Paleolithic period. Different studies using mitochondrial DNA, Y chromosome and SNP genotyping in Tunisia also showed the admixed and intermediate genetic architecture of the Tunisian population [63–67].

Haplotype analysis of the selected breast cancer susceptibility loci, showed that at risk haplotypes on 2p24, 4q21, 6q25, 9q31, 10q26,11p15, 11q13 and 14q32 loci are considerably frequent in the Tunisian population (> 20%). When comparing the allele frequencies of several common variants between Tunisian and other ethnic groups, some variants found on these loci showed significant differences between populations. Indeed, 6q25-rs2046210 and 14q32-rs941764, differ significantly between Tunisians and all other population expect ASW and MEX populations, respectively. This represents an argument of the admixed nature of the Tunisian population.

The 6q25 represents an ambiguous locus in breast cancer association studies in different ethnic groups [21]. Indeed, rs2046210 was found to be associated with breast cancer risk in European and East Asian populations but not in African populations. However, subsequent studies showed that other variants on the 6q25 region are associated with breast cancer risk in Africans namely: rs9397435 and rs2046211 [68]. Interestingly, the 6q25 locus contains the *ESR1* gene that encodes for the estrogen receptor protein which is strongly involved in breast carcinogenesis. Thus, association studies of the 6q25 SNPs in the North African population may help to explain the development of specific breast cancer phenotypes in North African population such as the triple negative breast cancer subtype.

Moreover, two variants (rs1219648 and rs2981582) found on the 10q26 locus have been recently identified as associated with breast cancer risk in the Tunisian population [54]. These two variants seem to be in a strong correlation (r^2^ = 0.8, Additional file 2: Figure S5.) meaning that they are carried by the same haplotype. Therefore, further analysis on the 10q26 locus in the TUN population should be prioritized.

In addition, the frequency of rs13329835 seems to differ significantly between Tunisians and all other HapMap populations. Recent studies showed that this variant is associated with breast cancer risk in Europeans and Afro Americans [24, 69]. Because, the allele frequency for this SNP differ considerably across ethnic groups, association studies in the Tunisian population are required to assess its association with breast cancer risk.

We also performed in silico functional analysis in order to identify SNPs more or less likely to have functional effects. High RegulomeDB scores were assigned to variants on the 4q21, 19p13 and 17q21 loci.

The functional impact of rs1494961 and rs11099601 at 4q21 locus have been previously investigated by our group using eQTL analysis and ENCODE data. Significant eQTL associations and interactions with different biofeatures such as histones and enhancer elements have been identified for these two SNPs [70].

Fine-scale mapping studies have been also performed on the 19p13 locus in order to identify causal variants at this breast cancer susceptibility locus [71]. In this current study, 19p13-rs4808801 was identified as a putative functional SNP with interesting functional evidence (eQtl association with *SSBP4* gene, *p* = 1.6 × 10^− 5^). Our data also showed that rs4808801 is the variant that contributed more to the inter-ethnic genetic diversity between African and non-African-populations.

However, no functional studies have been reported for 17q21-rs9911630 except one of our previous studies that mentioned the potential association of this variant with differential allelic expression [62].

Here, we identified rs9911630-*BRCA1* variant as a haplogroup tagging SNP in the Tunisian population. Statistical analysis also showed that the frequency of this variant varies significantly between ancient populations (Africans) and more recent ones (Europeans and Asians). rs9911630 frequency in Tunisia seems to be between that of Europeans and Africans which would be expected in this admixed population with European and African heritage. eQTL analysis indicated that rs9911630 is significantly associated with the expression levels of *NBR2* and *BRCA1* genes. Significant eQTls were also observed between rs9911630 and other genes namely: *CTD-3199 J23.6* and *LINC00854.*

The observed eQTLs associated between rs9911630 and *BRCA1* derived from analysis performed in fibroblast, lymphoblastoid cell line and T cell. However, eQTL associations between this variant and *NBR2, CTD-3199 J23.6* and *LINC00854* derived from breast mammary cell lines. As regulatory effects are often tissue-specific this may explain the different eQTL associations observed in these different cell types. *CTD-3199 J23.6* is a transcribed processed pseudogene, no further information on it is function or its involvement in carcinogenesis have been reported. *LINC00854* is an RNA gene, and is affiliated with long non-coding RNA class (lncRNAs). *NBR2* is also a non-protein coding gene that encodes a long non-coding RNA and suppresses tumor development through regulation of adenosine monophosphate–activated protein kinase (AMPK) activation [72]. It resides adjacent to the tumor suppressor gene *BRCA1*. Given the close proximity of the human *BRCA1* and *NBR2* genes, it has been suggested that these 2 genes may be coordinately expressed. Since many lncRNAs regulate the transcription of neighboring genes, the hypothesis that *NBR2* regulates *BRCA1* transcription is also plausible [73].

lncRNAs is still a poorly understood class of non-coding RNAs that refer to a classified group of RNA transcripts longer than 200 nucleotides and have no apparent protein-coding potential. Accumulating recent evidence links long non-coding RNAs to cancer metabolism by regulating various aspects of cancer metabolism through their cross-talk with other macromolecules [74, 75]. In addition, recent studies showed that lncRNAs can cross-talk with other non-coding RNAs such as microRNAs through competing endogenous RNA (ceRNA) mechanisms [76]. Interestingly, our results indicated that rs9911630 alters the binding sites of three non-human miRNAs (miR-2766, bmo-miR3287 and ssa-miR-19d-5p). The regulation of gene expression by cross-species microRNAs has been previously reported and their link to cancer development or prevention have been also explored [77]. Indeed, non-human microRNA such as plant and animal derived microRNAs have been detected in human blood in a large nutrigenomics study cohort [77]. Moreover, the role of food-related microRNAs in regulating the expression of key human cancer-related genes was highly debated [78, 79]. Thus, long and small non coding RNAs (lncRNAs and microRNAs), altogether with protein-coding RNAs may form complex regulatory networks involved in various aspects of cancer biology.

Interestingly, significant eQTL associations observed between rs9911630 and *BRCA1* have been observed only in European and Asian individuals but not in Africans and the allele frequencies of this variants also differ significantly between African and non-African populations. We hypothesized that differences in *BRCA1* expression levels between African and non African populations may be explained by difference in the allele frequency of this variants and by differences in the expression level of some exogenous microRNAs mainly due to different environmental and lifestyle factors such as food intake habits. Moreover, in a separate study, we identified rs16942-*BRCA1* as a modifier variant of breast cancer risk in *BRCA1* mutation carriers [80]. rs16942 seems to be in complete LD with rs9911630 in populations from European origins but not in African populations. Thus, we suggest that rs9911630 is a potential functional variant that may be associated with breast cancer risk in an ethnic specific manner by altering the expression level of key tumor suppressor genes such as *BRCA1* and *NBR2*. However, the association of this variant with breast cancer risk in different ethnic groups warrants further association and functional investigations.

## Conclusions

The goal of this research was to explore the genetic architecture of a number of breast cancer risk SNPs in the Tunisian population comparing their frequency to 11 different ethnic groups. The observed discordance in the genetic background between populations highlights the necessity for researchers to establish a specific genotype profile for each population. Therefore, caution should be exercised in applying any genetic risk prediction model based on tagSNPs outside of the ancestry group in which it was derived. To our best knowledge, this study is the largest investigation of breast cancer common loci in the general Tunisian population. Further investigations are in progress by our group regarding the association of the potential functional variants identified in this study and breast cancer risk in the Tunisian population.

## Additional files

Additional file 1: **Table S1** Breast cancer loci and variants investigated in the Tunisian population. **Table S2** Data sources for in silico analyses of variants with high RegulomeDB scores. **Table S3** Allelic frequency of the selected breast cancer polymorphisms and comparison of these frequencies between Tunisian and HapMap populations (Pairwise *pvalues* < 0.05). *Polymorphisms highlighted in grey are the four SNPs that showed an allelic frequency significantly different between Tunisians and all other HapMap populations. **Table S4** MicroRNA binding sites altered by the *BRCA1*-rs9911630 variant. (DOCX 410 kb)

Additional file 2: **Figure S1** GTEX Boxplots representing the most significant eQTL results for variant rs9911630 in breast mammary tissue. Box plots represent the expression levels of the indicated transcripts with respect to the rs9911630 genotypes. Expression levels are shown for **(a)** *NBR2* gene, **(b)** *CTD-3199 J23.6* gene and **(c)** *LINC00910* gene. Horizontal bars indicate mean expression level per genotype. Additional information on the eQTL *p* values are reported in Table 1. **Figure S2** Alignment of the sequence around rs9911630 with binding site of **(a)** bmo-miR-3287, **(b)** ssa-miR-19d-5p and **(c)** mse-miR-2766. The SNP is shown in red and the allele binding the microRNA is also shown. **Figure S3** Contributions of variables (SNPs and populations) in Dim1 and Dim2. The contribution of each tested variable (based on their variants frequencies) in the general variability between the different selected populations shown on the PCA (a) to the first dimension of the PCA (Dim1 for the variability between African and non-African populations –see Fig. 3) and (b) to the second dimension of the PCA (Dim2 for the variability between European and non-European populations). **Figure S4** Contributions of each tested population in the general variability between the different selected populations shown on the PCA **(a)** the first dimension of the PCA (Dim1) and **(b)** the second dimension of the PCA (Dim2). **Figure S5** A map of the linkage disequilibrium in intron 2 of *FGFR2* gene containing two SNPs associated with breast cancer risk in the Tunisian population (rs1219648 and rs2981582). (DOCX 263 kb)

## Abbreviations

ASW: African ancestry in Southwest USA
BC: Breast Cancer
CEU: Utah residents with Northern and Western European ancestry from the CEPH collection
CHB: Han Chinese in Beijing, China
CHD: Chinese in Metropolitan Denver, Colorado
ENCODE: ENCyclopedia Of DNA elements
Genevar: GENe Expression VARiation
GIH: Gujarati Indians in Houston, Texas
GMAF: Global Minor Allele Frequency
Haplogroup: Haplotype groups
htSNPs: Haplotype tag SNPs
HW: Hardy Weinberg
HWE: Hardy-weinberg equilibrium
IBS: Identity-by-state
JPT: Japanese in Tokyo, Japan
LD: Linkage disequilibrium
LWK: Luhya in Webuye, Kenya
MAF: Minor allele frequency
MDS: MultiDimensional scaling
MENA: North African and Middle East
MEX: Mexican ancestry in Los Angeles, California
MKK: Maasai in Kinyawa, Kenya
PCA: Principal components analysis
SNP: Single nucleotide polymorphism
TSI: Toscans in Italy
TUN: Tunisian population
YRI: Yoruba in Ibadan, Nigeria
