# Supplementary material for: A genome wide SNP genotyping study in the Tunisian population: specific reporting on a subset of common breast cancer risk loci
Source: BMC Cancer. 2018 Dec 29;18:1295. doi: 10.1186/s12885-018-5133-8 (PMC6310952; doi:10.1186/s12885-018-5133-8)
Supplement: Supplementary file 1 — Table S1 Breast cancer loci and variants investigated in the Tunisian population. Table S2 Data sources for in silico analyses of variants with high RegulomeDB scores. Table S3 Allelic frequency of the selected breast cancer polymorphisms and comparison of these frequencies between Tunisian and HapMap populations (Pairwise pvalues < 0.05). *Polymorphisms highlighted in grey are the four SNPs that showed an allelic frequency significantly different between Tunisians and all other HapMap populations. Table S4 MicroRNA binding sites altered by the BRCA1-rs9911630 variant. (DOCX 410 kb) [file 12885_2018_5133_MOESM1_ESM.docx]

**Table S1.** Breast cancer loci and variants investigated in the Tunisian population

| **Loci** | **Studied Genomic regions (Assembly GRCh37.p13)** | **Genes** | **Variants** | **References** |
| --- | --- | --- | --- | --- |
| **High penetrance mutations** | |  |  |  |
| 17q21 | chr17: 41,143,452-41,377,500 | *BRCA1* |  | [[1](#_ENREF_1)] |
| 13q12.3 | chr13: 32,845,093-33,053,652 | *BRCA2* |  | [[2](#_ENREF_2)] |
| 17p13.1 | chr17: 7,471,720-7,690,868 | *TP53* |  | [[3](#_ENREF_3)] |
| 10q23.3 | chr10: 89,523,195-89,828,532 | *PTEN* |  | [[4](#_ENREF_4)] |
| 19p13.3 | chr19: 1,105,798-1,328,434 | *STK11* |  | [[5](#_ENREF_5)] |
| 16q22.1 | chr16: 68,671,195-68,969,444 | *CDH1* |  | [[6](#_ENREF_6)] |
| **Moderate penetrance variants** | |  |  |  |
| 11q22.3 | chr11: 108,075,402-108,286,638 | *ATM* |  | [[7](#_ENREF_7), [8](#_ENREF_8)] |
| 22q12.1 | chr22: 28,983,731-29,237,822 | *CHEK2* |  | [[9](#_ENREF_9)] |
| 17q22-q24 | chr17: 59,656,547-60,040,920 | *BRIP1* |  | [[10](#_ENREF_10)] |
| 16p12.1 | chr16: 23,514,483-23,752,678 | *PALB2* |  | [[11](#_ENREF_11)] |
| **Lowpenetrance variants** | |  |  |  |
| 1p11 | Chr1: 121260613- 121300613 | *NOTCH2/FCGR1B* | rs11249433 | [[12](#_ENREF_12)] |
| 1p13 | Chr1: 114428389-114468389 | *TPN22/BCL2L15* | rs11552449 | [[13](#_ENREF_13)] |
| 1p36 | Chr1: 10546215- 10586215 | *PEX14* | rs616488 | [[13](#_ENREF_13)] |
| 1q32 | Chr1: 204498842-204538842 | *MDM4* | rs4245739 | [[14](#_ENREF_14)] |
|  | Chr1: 202167176- 202207176 | *LGR6* | rs6678914 | [[14](#_ENREF_14)] |
| 2p24.1 | Chr2: 19300803- 19340803 | *None* | rs12710696 | [[14](#_ENREF_14)] |
| 2q14.2 | Chr2: 121225122- 121265122 | *None* | rs4849887 | [[13](#_ENREF_13)] |
| 2q31.1 | Chr2: 174192894- 174232894 | *CDCA7* | rs1550623 | [[13](#_ENREF_13)] |
| 2q33.1 | Chr2: 202129589- 202169589 | *CASP8* | rs1045485 | [[13](#_ENREF_13)] |
|  | Chr2: 202123928- 202163928 | *IGFBP2, TPN2* | rs10931936 | [[15](#_ENREF_15)] |
| 2q35 | Chr2: 17885832- 17925832 | *DIRC3* | rs13387042 | [[16](#_ENREF_16)] |
|  | Chr2: 218276508- 218316508 |  | rs16857609 | [[13](#_ENREF_13)] |
| 3p24.1 | Chr3: 27396013- 27436013 | *SLC4A7/NEK10* | rs4973768 | [[17](#_ENREF_17)] |
|  | Chr3: 30662939- 30702939 | *TGFBR2* | rs12493607 | [[13](#_ENREF_13)] |
| 3p26.1 | Chr3: 4722276- 4762276 | *ITPR1/EGOT* | rs6762644 | [[13](#_ENREF_13)] |
| 4q21.23 | Chr4: 84362763- 84402763 | *HelQ/FAM175A/MRPS18C* | rs11099601 | [[18](#_ENREF_18)] |
|  | Chr4 :84394480-84354480 | *HelQ/FAM175A/MRPS18C* | rs1494961 | [[18](#_ENREF_18)] |
| 4q24 | Chr4: 106064778- 106104778 | *TET2* | rs9790517 | [[13](#_ENREF_13)] |
| 4q34.1 | Chr4: 175826426- 175866426 | *ADAM29* | rs6828523 | [[13](#_ENREF_13)] |
| 5p12 | Chr5: 44686498- 44726498 | *MRPS30/HCN1* | rs10941679 | [[12](#_ENREF_12)] |
| 5p15.33 | Chr5: 1259790- 1299790 | *TERT/CLPTM1L* | rs10069690 | [[13](#_ENREF_13)] |
| 5q11.2 | Chr5:56011884- 56051884 | *MAP3K1/MEIR3* | rs889312 | [[19](#_ENREF_19)] |
|  | Chr5: 58164061- 58204061 | *RAB3C* | rs10472076 | [[13](#_ENREF_13)] |
|  | Chr5: 58317481- 58357481 | *PDE4D* | rs1353747 | [[13](#_ENREF_13)] |
| 5q33.3 | Chr5: 158224083- 158264083 | *EBF1* | rs1432679 | [[13](#_ENREF_13)] |
| 6p23 | Chr6: 13702523- 13742523 | *RANBP9* | rs204247 | [[13](#_ENREF_13)] |
| 6p25.3 | Chr6: 1298878- 1338878 | *FOXQ1* | rs11242675 | [[13](#_ENREF_13)] |
| 6q14.1 | Chr6: 82173109- 82213109 | *None* | rs17530068 | [[13](#_ENREF_13)] |
| 6q25.1 | Chr6: 151894113- 151934113 | *ESR1/CCDC170* | rs3757318 | [[20](#_ENREF_20)] |
|  | Chr6: 151928366- 151968366 | *ESR1* | rs2046210 | [[13](#_ENREF_13)] |
| 7q35 | Chr7: 144054929- 144094929 | *ARHGEF5/NOBOX* | rs720475 | [[13](#_ENREF_13)] |
| 8p12 | Chr8: 29489616- 29529616 | *None* | rs9693444 | [[13](#_ENREF_13)] |
| 8q21.11 | Chr8: 76210301- 76250301 | *None* | rs6472903 | [[13](#_ENREF_13)] |
| 8q21.11 | Chr8: 76397937- 76437937 | *HNF4G* | rs2943559 | [[13](#_ENREF_13)] |
| 8q24.21 | Chr8: 128335618- 128375618 | *MYC/CASC21* | rs13281615 | [[19](#_ENREF_19)] |
| 8q24.21 | Chr8: 128367852- 128407852 | *MYC/CASC21* | rs1562430 | [[15](#_ENREF_15)] |
|  | Chr8: 129174641- 129214641 | *MIR1208* | rs11780156 | [[13](#_ENREF_13)] |
| 9p21.3 | Chr9: 22042134- 22082134 | *CDKN2A/B* | rs1011970 | [[15](#_ENREF_15)] |
| 9q31.2 | Chr9: 110868478- 110908478 | *KLF4/RAD23B* | rs865686 | [[21](#_ENREF_21)] |
|  | Chr9: 110286115- 110326115 | *None* | rs10759243 | [[13](#_ENREF_13)] |
| 10p12.31 | Chr10: 22012942- 22052942 | *MLLT10/DNAJC1* | rs7072776 | [[13](#_ENREF_13)] |
| 10p15.1 | Chr10: 5866734- 5906734 | *ANKRD16* | rs2380205 | [[15](#_ENREF_15)] |
| 10q21.2 | Chr10: 64258682- 64298682 | *ZNF365* | rs10995190 | [[15](#_ENREF_15)] |
| 10q22.3 | Chr10: 80821148- 80861148 | *ZMIZ1* | rs704010 | [[15](#_ENREF_15)] |
| 10q25.2 | Chr10: 114753927- 114793927 | *TCF7L2* | rs7904519 | [[13](#_ENREF_13)] |
| 10q26 | Chr10: 123332317- 123372317 | *FGFR2* | rs2981582 | [[19](#_ENREF_19)] |
| 10q26.13 | Chr10: 123317335- 123357335 | *FGFR2* | rs2981579 | [[13](#_ENREF_13)] |
| 10q26.12-q26.13 | Chr10: 123073901- 123113901 | *None* | rs11199914 | [[13](#_ENREF_13)] |
| 11p15.5 | Chr11: 1889006- 1929006 | *LSP1/H19* | rs3817198 | [[19](#_ENREF_19)] |
|  | Chr11: 1921946- 1981946 | *LSP1/H19* | rs909116 | [[15](#_ENREF_15)] |
| 11q13.3 | Chr11: 69308764- 69348764 | *CCND1/FGFs* | rs614367 | [[15](#_ENREF_15)] |
| 11q13.1 | Chr11: 65563066- 65603066 | *OVOL1* | rs3903072 | [[13](#_ENREF_13)] |
| 11q24 | Chr11: 129441171- 129481171 | *None* | rs11820646 | [[13](#_ENREF_13)] |
| 12p11 | Chr12: 28135080- 28175080 | *PTHLH* | rs10771399 | [[22](#_ENREF_22)] |
| 12p13 | Chr12: 14393931- 14433931 | *None* | rs12422552 | [[13](#_ENREF_13)] |
| 12q22 | Chr12: 96007759- 96047759 | *NTN4* | rs17356907 | [[13](#_ENREF_13)] |
| 12q24 | Chr12: 115816522- 115856522 | *TBX3/MAPKAP5* | rs1292011 | [[22](#_ENREF_22)] |
| 13q13 | Chr13: 32952626- 32992626 | *BRCA2* | rs11571833 | [[13](#_ENREF_13)] |
| 14q13 | Chr14: 37112769- 37152769 | *PAX9/SLC25A21* | rs2236007 | [[13](#_ENREF_13)] |
| 14q24 | Chr14: 69014682- 69054682 | *RAD51B* | rs999737 | [[12](#_ENREF_12)] |
|  | Chr14: 69019588- 69059588 | *RAD51B* | rs8009944 | [[15](#_ENREF_15)] |
|  | Chr14: 68640428- 68680428 | *RAD51L1* | rs2588809 | [[13](#_ENREF_13)] |
| 14q32 | chr14: 91821246-91857157 | *CCDC88C* | rs941764 | [[13](#_ENREF_13)] |
| 16q12.1 | Chr16: 52528037- 52568037 | *TOX3/LOC643714* | rs12443621 | [[23](#_ENREF_23)] |
|  | Chr16: 52566341- 52586341 | *TOX3/LOC643714* | rs3803662 | [[19](#_ENREF_19)] |
|  | Chr16 :52554167-52514167 | *TOX3/LOC643714* | rs8051542 | [[24](#_ENREF_24)] |
| 16q12.2 | Chr16: 53793367- 53833367 | *MIR1972-2-FTO* | rs17817449 | [[13](#_ENREF_13)] |
| 16q12.2 | Chr16: 53835291- 53875291 | *FTO* | rs11075995 | [[14](#_ENREF_14)] |
| 16q23.2 | Chr16: 80630805- 80670805 | *CDYL2* | rs13329835 | [[13](#_ENREF_13)] |
| 17q23 | Chr17: 53036471- 53076471 | *STXBP4/COX11* | rs6504950 | [[17](#_ENREF_17)] |
| 18q11 | Chr18: 24317424- 24357424 | *None* | rs527616 | [[13](#_ENREF_13)] |
|  | Chr18: 24550667- 24590667 | *CHST9* | rs1436904 | [[13](#_ENREF_13)] |
| 19p13 | Chr19: 17369704- 17409704 | *MERIT40* | rs8170 | [[13](#_ENREF_13)] |
|  | Chr19: 17374124- 17414124 | *MERIT40* | rs2363956 | [[25](#_ENREF_25)] |
|  | Chr19: 18551141- 18591141 | *SSBP4/ISYNA1/ELL* | rs4808801 | [[13](#_ENREF_13)] |
| 19q13 | Chr19: 44266513- 44306513 | *KCNN4/ZNF283* | rs3760982 | [[13](#_ENREF_13)] |
| 20q11 | Chr20: 32568095- 32608095 | *RALY* | rs2284378 | [[25](#_ENREF_25)] |
| 21q21 | Chr21: 16500832- 16540832 | *NRIP1* | rs2823093 | [[22](#_ENREF_22)] |
| 22q12 | Chr22: 29601477- 29641477 | *EMID1/RHBDD3* | rs132390 | [[13](#_ENREF_13)] |
| 22q13 | Chr22: 40846234- 40916234 | *MKL1* | rs6001930 | [[14](#_ENREF_14)] |

**References Table S1.**

1. Miki Y, Swensen J, Shattuck Eidens D, Futreal PA, Harshman K, Tavtigian S, Liu Q, Cochran C, Bennett LM, Ding W *et al*: **A strong candidate for the breast and ovarian cancer susceptibility gene BRCA1**. *Science (New York, NY)* 1994, **266**(5182):66 71.

2. Wooster R, Bignell G, Lancaster J, Swift S, Seal S, Mangion J, Collins N, Gregory S, Gumbs C, Micklem G: **Identification of the breast cancer susceptibility gene BRCA2**. *Nature* 1995, **378**(6559):789 792.

3. Borresen AL, Andersen TI, Garber J, Barbier Piraux N, Thorlacius S, Eyfjord J, Ottestad L, Smith Sorensen B, Hovig E, Malkin D *et al*: **Screening for germ line TP53 mutations in breast cancer patients**. *Cancer research* 1992, **52**(11):3234 3236.

4. Lynch ED, Ostermeyer EA, Lee MK, Arena JF, Ji H, Dann J, Swisshelm K, Suchard D, MacLeod PM, Kvinnsland S *et al*: **Inherited mutations in PTEN that are associated with breast cancer, cowden disease, and juvenile polyposis**. *American journal of human genetics* 1997, **61**(6):1254 1260.

5. Mehenni H, Resta N, Park JG, Miyaki M, Guanti G, Costanza MC: **Cancer risks in LKB1 germline mutation carriers**. *Gut* 2006, **55**(7):984 990.

6. Kanai Y, Oda T, Tsuda H, Ochiai A, Hirohashi S: **Point mutation of the E cadherin gene in invasive lobular carcinoma of the breast**. *Japanese journal of cancer research : Gann* 1994, **85**(10):1035 1039.

7. Tavtigian SV, Oefner PJ, Babikyan D, Hartmann A, Healey S, Le Calvez Kelm F, Lesueur F, Byrnes GB, Chuang SC, Forey N *et al*: **Rare, evolutionarily unlikely missense substitutions in ATM confer increased risk of breast cancer**. *American journal of human genetics* 2009, **85**(4):427 446.

8. Renwick A, Thompson D, Seal S, Kelly P, Chagtai T, Ahmed M, North B, Jayatilake H, Barfoot R, Spanova K *et al*: **ATM mutations that cause ataxia telangiectasia are breast cancer susceptibility alleles**. *Nature genetics* 2006, **38**(8):873 875.

9. Meijers Heijboer H, Wijnen J, Vasen H, Wasielewski M, Wagner A, Hollestelle A, Elstrodt F, van den Bos R, de Snoo A, Fat GT *et al*: **The CHEK2 1100delC mutation identifies families with a hereditary breast and colorectal cancer phenotype**. *American journal of human genetics* 2003, **72**(5):1308 1314.

10. Ren LP, Xian YS, Diao DM, Chen Y, Guo Q, Dang CX: **Further evidence for the contribution of the BRCA1 interacting protein terminal helicase 1 (BRIP1) gene in breast cancer susceptibility**. *Genetics and molecular research : GMR* 2013, **12**(4):5793 5801.

11. Erkko H, Xia B, Nikkila J, Schleutker J, Syrjakoski K, Mannermaa A, Kallioniemi A, Pylkas K, Karppinen SM, Rapakko K *et al*: **A recurrent mutation in PALB2 in Finnish cancer families**. *Nature* 2007, **446**(7133):316 319.

12. Thomas G, Jacobs KB, Kraft P, Yeager M, Wacholder S, Cox DG, Hankinson SE, Hutchinson A, Wang Z, Yu K *et al*: **A multistage genome wide association study in breast cancer identifies two new risk alleles at 1p11.2 and 14q24.1 (RAD51L1)**. *Nature genetics* 2009, **41**(5):579 584.

13. Michailidou K, Hall P, Gonzalez Neira A, Ghoussaini M, Dennis J, Milne RL, Schmidt MK, Chang Claude J, Bojesen SE, Bolla MK *et al*: **Large scale genotyping identifies 41 new loci associated with breast cancer risk**. *Nature genetics* 2013, **45**(4):353 361, 361e351 352.

14. Garcia Closas M, Couch FJ, Lindstrom S, Michailidou K, Schmidt MK, Brook MN, Orr N, Rhie SK, Riboli E, Feigelson HS *et al*: **Genome wide association studies identify four ER negative specific breast cancer risk loci**. *Nature genetics* 2013, **45**(4):392 398, 398e391 392.

15. Turnbull C, Ahmed S, Morrison J, Pernet D, Renwick A, Maranian M, Seal S, Ghoussaini M, Hines S, Healey CS *et al*: **Genome wide association study identifies five new breast cancer susceptibility loci**. *Nature genetics* 2010, **42**(6):504 507.

16. Stacey SN, Manolescu A, Sulem P, Rafnar T, Gudmundsson J, Gudjonsson SA, Masson G, Jakobsdottir M, Thorlacius S, Helgason A *et al*: **Common variants on chromosomes 2q35 and 16q12 confer susceptibility to estrogen receptor positive breast cancer**. *Nature genetics* 2007, **39**(7):865 869.

17. Ahmed S, Thomas G, Ghoussaini M, Healey CS, Humphreys MK, Platte R, Morrison J, Maranian M, Pooley KA, Luben R *et al*: **Newly discovered breast cancer susceptibility loci on 3p24 and 17q23.2**. *Nature genetics* 2009, **41**(5):585 590.

18. Hamdi Y, Soucy P, Adoue V, Michailidou K, Canisius S, Lemacon A, Droit A, Andrulis IL, Anton Culver H, Arndt V *et al*: **Association of breast cancer risk with genetic variants showing differential allelic expression: Identification of a novel breast cancer susceptibility locus at 4q21**. *Oncotarget* 2016, **7**(49):80140 80163.

19. Easton DF, Pooley KA, Dunning AM, Pharoah PD, Thompson D, Ballinger DG, Struewing JP, Morrison J, Field H, Luben R *et al*: **Genome wide association study identifies novel breast cancer susceptibility loci**. *Nature* 2007, **447**(7148):1087 1093.

20. Zheng W, Long J, Gao YT, Li C, Zheng Y, Xiang YB, Wen W, Levy S, Deming SL, Haines JL *et al*: **Genome wide association study identifies a new breast cancer susceptibility locus at 6q25.1**. *Nature genetics* 2009, **41**(3):324 328.

21. Fletcher O, Johnson N, Orr N, Hosking FJ, Gibson LJ, Walker K, Zelenika D, Gut I, Heath S, Palles C *et al*: **Novel breast cancer susceptibility locus at 9q31.2: results of a genome wide association study**. *Journal of the National Cancer Institute* 2011, **103**(5):425 435.

22. Ghoussaini M, Pharoah PD, Easton DF: **Inherited genetic susceptibility to breast cancer: the beginning of the end or the end of the beginning?***The American journal of pathology* 2013, **183**(4):1038 1051.

23. Woolcott CG, Maskarinec G, Haiman CA, Verheus M, Pagano IS, Le Marchand L, Henderson BE, Kolonel LN: **Association between breast cancer susceptibility loci and mammographic density: the Multiethnic Cohort**. *Breast cancer research : BCR* 2009, **11**(1):R10.

24. Bolton KL, Tyrer J, Song H, Ramus SJ, Notaridou M, Jones C, Sher T, Gentry Maharaj A, Wozniak E, Tsai YY *et al*: **Common variants at 19p13 are associated with susceptibility to ovarian cancer**. *Nature genetics* 2010, **42**(10):880 884.

25. Siddiq A, Couch FJ, Chen GK, Lindstrom S, Eccles D, Millikan RC, Michailidou K, Stram DO, Beckmann L, Rhie SK *et al*: **A meta analysis of genome wide association studies of breast cancer identifies two novel susceptibility loci at 6q14 and 20q11**. *Human molecular genetics* 2012, **21**(24):5373 5384.

| **Table S2.** Data sources for *in silico* analyses of variants with high RegulomeDB scores**.** | | | | | |
| --- | --- | --- | --- | --- | --- |
| **a) rs1494961(chr4:** [**84374480**](https://www.ncbi.nlm.nih.gov/variation/view/?q=rs1494961&filters=source:dbsnp&assm=GCF_000001405.25)**) (RegulomeDB score 1f)** | | | | | |
|  | **Fixed transcription factor or affected gene** | **Location** | **Method** | **Cell Type/Tissue** | **Reference** |
| **Motifs** | AP-1 | chr4:84374469..84374480 | Footprinting | Huvec | [[1](#_ENREF_1)] |
|  | Rfx3 | chr4:84374479..84374502 | PWM | NA | [[2](#_ENREF_2)] |
|  | MAF | chr4:84374470..84374481 | PWM | NA | [[3](#_ENREF_3)] |
|  | AP-1 | chr4:84374469..84374480 | PWM | NA | [[3](#_ENREF_3)] |
| **eQTL** | *MRPS18C* | chr4:84374479..84374480 | eQTL | Monocytes | [[2](#_ENREF_2)] |
| **Chromatin structure** |  | chr4:84374420..84374570 | DNase-seq | Hmvecdblad | Encode (Encyclopedia of DNA Elements project) |
|  |  | chr4:84374420..84374570 | DNase-seq | Hrgec |  |
|  |  | chr4:84374460..84374610 | DNase-seq | Hmvecdblad |  |
| **Histone modification** |  | chr4:84373400..84374800 | ChromHMM | Ovary | REMC (Roadmap Epigenomics Mapping Consortium) |
|  |  | chr4:84330800..84374800 | ChromHMM | HeLa-S3 Cervical Carcinoma Cell Line |  |
|  |  | chr4:84374400..84374800 | ChromHMM | Breast MyoepithelialPrimaryCells |  |
|  |  | chr4:84372000..84374800 | ChromHMM | HMEC Mammary Epithelial Primary Cells |  |
|  |  | chr4:84373600..84375000 | ChromHMM | Breast variant Human Mammary Epithelial Cells (vHMEC) |  |

**PWM**: Position Weight Matrices

**ChromHMM**: Chromatine Hidden Markov Model

| **Table S2.** Data sources for in silico analyses of variants with high RegulomeDB scores (continued) | | | | | |
| --- | --- | --- | --- | --- | --- |
| **b) rs11099601 (Chr4:** [**84382763**](https://www.ncbi.nlm.nih.gov/variation/view/?q=rs11099601&filters=source:dbsnp&assm=GCF_000001405.25)**) ( RegulomeDB score 1f)** | | | | | |
|  | **Affected gene** | **Target Location**  **(Assembly NCBI38/hg19)** | **Method** | **Cell Type/Tissue** | **Reference** |
| **eQTL** | *MRPS18C* | chr4:84382762..84382763 | eQTL | Monocytes | [[4](#_ENREF_4)] |
| **Chromatin structure** |  | chr4:84382700..84382850 | DNase-seq | K562 | Encode |
|  |  | chr4:84382700..84382850 | DNase-seq | K562 | Encode |
|  |  | chr4:84382459..84382776 | FAIRE | MCF7 | Encode |
| **Histone modification** |  | chr4:84377800..84386600 | ChromHMM | Ovary | REMC |
|  |  | chr4:84381000..84391400 | ChromHMM | HeLa-S3 Cervical Carcinoma Cell Line |  |
|  |  | chr4:84379000..84390800 | ChromHMM | Breast Myoepithelial Primary Cells |  |
|  |  | chr4:84378800..84384000 | ChromHMM | Breast variant Human Mammary Epithelial Cells (vHMEC) |  |

| **Table S2.** Data sources for *in silico* analyses of variants with high RegulomeDB scores (continued) | | | | | |
| --- | --- | --- | --- | --- | --- |
| **c) rs9911630 (chr17:** [**41188342**](https://www.ncbi.nlm.nih.gov/variation/view/?q=rs9911630&filters=source:dbsnp&assm=GCF_000001405.25)) **( RegulomeDB score 1b)** | | | | | |
|  | **Fixed transcription factor or affected gene** | **Target position** | **Method** | **Cell Type/Tissue** | **Reference** |
| **Transcription factors affected by the variant** | POLR2A | chr17:41188282..41188758 | Chip seq | H1-hESC | Encode |
|  | ZNF143 | chr17:41188256..41188546 |  |  |  |
|  | MAFK | chr17:41188269..41188559 |  |  |  |
|  | SP1 | chr17:41188312..41188636 |  |  |  |
|  | TBP | chr17:41188280..41188656 |  |  |  |
|  | YY1 | chr17:41188307..41188643 |  |  |  |
| **Motifs** | Pax-4 | chr17:41188326..41188347 | Footprinting | H1hesc | [[1](#_ENREF_1)] |
|  | Pax-4 | chr17:41188326..41188347 | PWM | NA | [[3](#_ENREF_3)] |
| **eQTL** | *TMEM106A* | chr17:41188341..41188342 | eQTL | Lymphoblastoid | [[5](#_ENREF_5)] |
|  | *BRCA1* | chr17:41188341..41188342 |  | Monocytes | [[2](#_ENREF_2)] |
| **Histone modification** |  | chr17:41177800..41194600 | ChromHMM | Ovary | REMC |
|  |  | chr17:41182200..41201000 | ChromHMM | HeLa-S3 Cervical Carcinoma Cell Line |  |
|  |  | chr17:41177600..41193000 | ChromHMM | HMEC Mammary Epithelial Primary Cells |  |
|  |  | chr17:41182400..41193600 | ChromHMM | Breast variant Human Mammary Epithelial Cells (vHMEC) |  |

| **Table S2.** Data sources for *in silico* analyses of variants with high RegulomeDB scores (continued) | | | | | |
| --- | --- | --- | --- | --- | --- |
| **d) rs4808801(chr19:** [**18571141**](https://www.ncbi.nlm.nih.gov/variation/view/?q=rs4808801&filters=source:dbsnp&assm=GCF_000001405.25)**) ( RegulomeDB score 1f)** | | | | | |
|  | **Fixed transcription factor or affected gene** | **Location** | **Method** | **Cell Type/Tissue** | **Reference** |
| **Transcription factors affected by the variant** | TFAP2A | chr19:18570669..18571199 | ChIP-seq | HeLa-S3 | Encode |
| **eQTL** | *ELL* | chr19:18571140..18571141 | eQTL | Monocytes | [[4](#_ENREF_4)] |
| **Chromatin Structure** |  | chr19:18570600..18571599 | DNase-seq | HMEC | Encode |
|  |  | chr19:18570351..18571181 | FAIRE | HelaS3 | Encode |
|  |  | chr19:18570518..18571540 | FAIRE | HelaS3 | Encode |
| **Histone modification** |  | chr19:18570800..18571800 | ChromHMM | Ovary | REMC |
|  |  | chr19:18570800..18571200 | ChromHMM | HeLa-S3 Cervical Carcinoma Cell Line |  |
|  |  | chr19:18570200..18572600 | ChromHMM | HMEC Mammary Epithelial Primary Cells |  |
|  |  | chr19:18570600..18573000 | ChromHMM | Breast variant Human Mammary Epithelial Cells (vHMEC) |  |
|  |  | chr19:18570000..18572800 | ChromHMM | Breast variant Human Mammary Epithelial Cells (vHMEC) |  |

**References Table S2.**

1. Pique-Regi R, Degner JF, Pai AA, Gaffney DJ, Gilad Y, Pritchard JK: **Accurate inference of transcription factor binding from DNA sequence and chromatin accessibility data**. *Genome research* 2011, **21**(3):447-455.

2. Zeller T, Wild P, Szymczak S, Rotival M, Schillert A, Castagne R, Maouche S, Germain M, Lackner K, Rossmann H *et al*: **Genetics and beyond--the transcriptome of human monocytes and disease susceptibility**. *PloS one* 2010, **5**(5):e10693.

3. Matys V, Kel-Margoulis OV, Fricke E, Liebich I, Land S, Barre-Dirrie A, Reuter I, Chekmenev D, Krull M, Hornischer K *et al*: **TRANSFAC and its module TRANSCompel: transcriptional gene regulation in eukaryotes**. *Nucleic acids research* 2006, **34**(Database issue):D108-110.

4. Badis G, Berger MF, Philippakis AA, Talukder S, Gehrke AR, Jaeger SA, Chan ET, Metzler G, Vedenko A, Chen X *et al*: **Diversity and complexity in DNA recognition by transcription factors**. *Science (New York, NY)* 2009, **324**(5935):1720-1723.

5. Veyrieras JB, Kudaravalli S, Kim SY, Dermitzakis ET, Gilad Y, Stephens M, Pritchard JK: **High-resolution mapping of expression-QTLs yields insight into human gene regulation**. *PLoS genetics* 2008, **4**(10):e1000214.

**Table S3.** Allelic frequency of the selected breast cancer polymorphisms and comparison of these frequencies between Tunisian and HapMap populations (Pairwise *pvalues*< 0.05)

*Polymorphisms highlighted in grey are the four SNPs that showed an allelic frequency significantly different between Tunisians and all other HapMap populations

| **rs ID** | **Population** | **Major allele** | **Major allele count** | **Major allelefrequency** | **Minor allele** | **Minor allele count** | **Minor allelefrequency** | **Major Homozygousgenotype count** | **Heterozygous genotype count** | **Minor homozygousgenotype count** | **Total genotype count** | **pairwise pvalue** |
| --- | --- | --- | --- | --- | --- | --- | --- | --- | --- | --- | --- | --- |
| rs4245739 (C) | CEU | A | 157 | 0,692 | C | 69 | 0,308 | 55 | 47 | 11 | 113 | 0.241 |
| rs4245739 (C) | TSI | A | 123 | 0,699 | C | 53 | 0,301 | 44 | 35 | 9 | 88 | 0.2431 |
| rs4245739 (C) | CHB | A | 160 | 0,952 | C | 8 | 0,048 | 76 | 8 | 0 | 84 | 2.601e13 |
| rs4245739 (C) | JPT | A | 161 | 0,947 | C | 9 | 0,053 | 76 | 9 | 0 | 85 | 5.95e13 |
| rs4245739 (C) | CHD | A | 169 | 0,994 | C | 1 | 0,006 | 84 | 1 | 0 | 85 | 2.2e16 |
| rs4245739 (C) | GIH | A | 136 | 0,773 | C | 40 | 0,227 | 51 | 34 | 3 | 88 | 0.004396 |
| rs4245739 (C) | MEX | A | 72 | 0,714 | C | 28 | 0,286 | 25 | 22 | 3 | 50 | 0.191 |
| rs4245739 (C) | ASW | A | 68 | 0,665 | C | 34 | 0,335 | 22 | 24 | 5 | 51 | 0.7298 |
| rs4245739 (C) | MKK | A | 212 | 0,77 | C | 66 | 0,23 | 84 | 44 | 11 | 139 | 0.002477 |
| rs4245739 (C) | LWK | A | 115 | 0,661 | C | 59 | 0,339 | 36 | 43 | 8 | 87 | 0.7392 |
| rs4245739 (C) | YRI | A | 186 | 0,831 | C | 38 | 0,169 | 77 | 32 | 3 | 112 | 4.094e06 |
| rs4245739 (C) | TUN | A | 173 | 0,667 | C | 97 | 0,333 | 57 | 59 | 19 | 135 |  |
| rs12710696 (T) | CEU | T | 112 | 0,637 | C | 82 | 0,363 | 31 | 50 | 16 | 113 | 0.1879 |
| rs12710696 (T) | TSI | T | 109 | 0,619 | C | 67 | 0,381 | 33 | 43 | 12 | 88 | 0.0316 |
| rs12710696 (T) | CHB | T | 126 | 0,75 | C | 42 | 0,25 | 44 | 38 | 2 | 84 | 1.129e06 |
| rs12710696 (T) | JPT | T | 116 | 0,674 | C | 56 | 0,326 | 37 | 42 | 7 | 86 | 0.001012 |
| rs12710696 (T) | CHD | T | 108 | 0,643 | C | 60 | 0,357 | 33 | 42 | 9 | 84 | 0.00924 |
| rs12710696 (T) | GIH | T | 68 | 0,386 | C | 108 | 0,614 | 12 | 44 | 32 | 88 | 0.01293 |
| rs12710696 (T) | MEX | T | 69 | 0,69 | C | 31 | 0,31 | 24 | 21 | 5 | 50 | 0.003074 |
| rs12710696 (T) | ASW | T | 42 | 0,396 | C | 64 | 0,604 | 11 | 20 | 22 | 53 | 0.05853 |
| rs12710696 (T) | MKK | T | 131 | 0,465 | C | 151 | 0,535 | 32 | 67 | 42 | 141 | 0.3129 |
| rs12710696 (T) | LWK | T | 77 | 0,428 | C | 103 | 0,572 | 20 | 37 | 33 | 90 | 0.1015 |
| rs12710696 (T) | YRI | T | 99 | 0,438 | C | 127 | 0,562 | 24 | 51 | 38 | 113 | 0.1255 |
| rs12710696 (T) | TUN | T | 138 | 0,489 | C | 132 | 0,511 | 31 | 76 | 28 | 135 |  |
| rs1550623 (G) | CEU | A | 192 | 0,845 | G | 34 | 0,155 | 82 | 28 | 3 | 113 | 0.0275 |
| rs1550623 (G) | TSI | A | 155 | 0,881 | G | 21 | 0,119 | 69 | 17 | 2 | 88 | 0.0039 |
| rs1550623 (G) | CHB | A | 90 | 76 | G | ND | ND | 45 | 0 | 0 | 45 | 1.766e06 |
| rs1550623 (G) | JPT | A | 169 | 0,983 | G | 3 | 0,017 | 83 | 3 | 0 | 86 | 1.262e09 |
| rs1550623 (G) | CHD | A | ND | ND | G | ND | ND | ND | ND | ND | ND | ND |
| rs1550623 (G) | GIH | A | 145 | 0,824 | G | 31 | 0,176 | 61 | 23 | 4 | 88 | 0.1839 |
| rs1550623 (G) | MEX | A | 80 | 0,8 | G | 20 | 0,2 | 32 | 16 | 2 | 50 | 0.5876 |
| rs1550623 (G) | ASW | A | 63 | 0,594 | G | 43 | 0,406 | 18 | 27 | 8 | 53 | 0.001308 |
| rs1550623 (G) | MKK | A | 206 | 0,72 | G | 80 | 0,28 | 75 | 56 | 12 | 143 | 0.2486 |
| rs1550623 (G) | LWK | A | 124 | 0,689 | G | 56 | 0,311 | 39 | 46 | 5 | 90 | 0.08478 |
| rs1550623 (G) | YRI | A | 148 | 0,655 | G | 78 | 0,345 | 52 | 44 | 17 | 113 | 0.008069 |
| rs1550623 (G) | TUN | A | 207 | 0,76 | G | 63 | 0,24 | 78 | 51 | 6 | 135 |  |
| rs1494961 (C) | CEU | T | 124 | 0,549 | C | 102 | 0,451 | 30 | 64 | 19 | 113 | 0.6778 |
| rs1494961 (C) | TSI | T | 78 | 0,448 | C | 96 | 0,552 | 16 | 46 | 25 | 87 | 0.1335 |
| rs1494961 (C) | CHB | T | 121 | 0,729 | C | 45 | 0,271 | 47 | 27 | 9 | 83 | 4.026e05 |
| rs1494961 (C) | JPT | T | 108 | 0,635 | C | 62 | 0,365 | 36 | 36 | 13 | 85 | 0.03106 |
| rs1494961 (C) | CHD | T | 112 | 0,659 | C | 58 | 0,341 | 38 | 36 | 11 | 85 | 0.008081 |
| rs1494961 (C) | GIH | T | 100 | 0,581 | C | 72 | 0,419 | 29 | 42 | 15 | 86 | 0.2963 |
| rs1494961 (C) | MEX | T | 66 | 0,66 | C | 34 | 0,34 | 23 | 20 | 7 | 50 | 0.02848 |
| rs1494961 (C) | ASW | T | 74 | 0,698 | C | 32 | 0,302 | 27 | 20 | 6 | 53 | 0.003473 |
| rs1494961 (C) | MKK | T | 216 | 0,755 | C | 70 | 0,245 | 76 | 64 | 3 | 143 | 2.774e08 |
| rs1494961 (C) | LWK | T | 151 | 0,839 | C | 29 | 0,161 | 61 | 29 | 0 | 90 | 1.781e11 |
| rs1494961 (C) | YRI | T | 189 | 0,836 | C | 37 | 0,164 | 79 | 31 | 3 | 113 | 5.58e13 |
| rs1494961 (C) | TUN | T | 142 | 0,474 | C | 128 | 0,526 | 38 | 66 | 31 | 113 |  |
| rs11099601 (C) | CEU | T | 124 | 0,554 | C | 100 | 0,446 | 30 | 64 | 18 | 112 | 0.601 |
| rs11099601 (C) | TSI | T | 78 | 0,443 | C | 98 | 0,557 | 16 | 46 | 26 | 88 | 0.09144 |
| rs11099601 (C) | CHB | T | 121 | 0,72 | C | 47 | 0,28 | 47 | 27 | 10 | 84 | 0.0001117 |
| rs11099601 (C) | JPT | T | 108 | 0,628 | C | 64 | 0,372 | 36 | 36 | 14 | 86 | 0.05298 |
| rs11099601 (C) | CHD | T | 111 | 0,653 | C | 59 | 0,347 | 37 | 37 | 11 | 85 | 0.01427 |
| rs11099601 (C) | GIH | T | 101 | 0,574 | C | 75 | 0,426 | 29 | 43 | 16 | 88 | 0.4122 |
| rs11099601 (C) | MEX | T | 66 | 0,66 | C | 34 | 0,34 | 23 | 20 | 7 | 50 | 0.03331 |
| rs11099601 (C) | ASW | T | 72 | 0,692 | C | 32 | 0,308 | 26 | 20 | 6 | 52 | 0.006246 |
| rs11099601 (C) | MKK | T | 216 | 0,755 | C | 70 | 0,245 | 76 | 64 | 3 | 143 | 4.495e08 |
| rs11099601 (C) | LWK | T | 151 | 0,839 | C | 29 | 0,161 | 61 | 29 | 0 | 90 | 2.889e11 |
| rs11099601 (C) | YRI | T | 189 | 0,836 | C | 37 | 0,164 | 79 | 31 | 3 | 113 | 9.742e13 |
| rs11099601 (C) | TUN | T | 143 | 0,466 | C | 127 | 0,534 | 38 | 67 | 30 | 135 |  |
| rs6828523 (A) | CEU | C | 206 | 0,912 | A | 20 | 0,088 | 93 | 20 | 0 | 113 | 0.008166 |
| rs6828523 (A) | TSI | C | 163 | 0,926 | A | 13 | 0,074 | 76 | 11 | 1 | 88 | 0.003859 |
| rs6828523 (A) | CHB | C | 124 | 0,747 | A | 42 | 0,253 | 47 | 30 | 6 | 83 | 0.06242 |
| rs6828523 (A) | JPT | C | 138 | 0,802 | A | 34 | 0,198 | 58 | 22 | 6 | 86 | 0.6176 |
| rs6828523 (A) | CHD | C | 115 | 0,676 | A | 55 | 0,324 | 41 | 33 | 11 | 85 | 0.000463 |
| rs6828523 (A) | GIH | C | 138 | 0,784 | A | 38 | 0,216 | 53 | 32 | 3 | 88 | 0.329 |
| rs6828523 (A) | MEX | C | 77 | 0,766 | A | 23 | 0,234 | 29 | 19 | 2 | 50 | 0.2845 |
| rs6828523 (A) | ASW | C | 72 | 0,679 | A | 34 | 0,321 | 25 | 22 | 6 | 53 | 0.002945 |
| rs6828523 (A) | MKK | C | 207 | 0,724 | A | 79 | 0,276 | 74 | 59 | 10 | 143 | 0.005534 |
| rs6828523 (A) | LWK | C | 103 | 0,572 | A | 77 | 0,428 | 32 | 39 | 19 | 90 | 6.898e09 |
| rs6828523 (A) | YRI | C | 124 | 0,549 | A | 102 | 0,451 | 35 | 54 | 24 | 113 | 3.851e11 |
| rs6828523 (A) | TUN | C | 223 | 0,821 | A | 47 | 0,179 | 89 | 45 | 1 | 135 |  |
| rs204247 (G) | CEU | A | 117 | 0,518 | G | 109 | 0,482 | 31 | 55 | 27 | 113 | 0.03706 |
| rs204247 (G) | TSI | A | 92 | 0,523 | G | 84 | 0,477 | 22 | 48 | 18 | 88 | 0.06772 |
| rs204247 (G) | CHB | A | 69 | 0,411 | G | 99 | 0,589 | 14 | 41 | 29 | 84 | 4.769e05 |
| rs204247 (G) | JPT | A | 66 | 0,384 | G | 106 | 0,616 | 12 | 42 | 32 | 86 | 3.389e06 |
| rs204247 (G) | CHD | A | 64 | 0,376 | G | 106 | 0,624 | 14 | 36 | 35 | 85 | 1.79e06 |
| rs204247 (G) | GIH | A | 106 | 0,602 | G | 70 | 0,398 | 32 | 42 | 14 | 88 | 0.8681 |
| rs204247 (G) | MEX | A | 57 | 0,57 | G | 43 | 0,43 | 18 | 21 | 11 | 50 | 0.5075 |
| rs204247 (G) | ASW | A | 71 | 0,683 | G | 33 | 0,317 | 26 | 19 | 7 | 52 | 0.2709 |
| rs204247 (G) | MKK | A | 196 | 0,685 | G | 90 | 0,315 | 64 | 68 | 11 | 143 | 0.0981 |
| rs204247 (G) | LWK | A | 146 | 0,811 | G | 34 | 0,189 | 60 | 26 | 4 | 90 | 1.562e05 |
| rs204247 (G) | YRI | A | 153 | 0,677 | G | 73 | 0,323 | 57 | 39 | 17 | 113 | 0.1785 |
| rs204247 (G) | TUN | A | 166 | 0,63 | G | 104 | 0,37 | 55 | 56 | 24 | 135 |  |
| rs2046210 (A) | CEU | G | 161 | 0,712 | A | 65 | 0,288 | 54 | 53 | 6 | 113 | 1.132e05 |
| rs2046210 (A) | TSI | G | 112 | 0,636 | A | 64 | 0,364 | 37 | 38 | 13 | 88 | 0.01503 |
| rs2046210 (A) | CHB | G | 109 | 0,649 | A | 59 | 0,351 | 35 | 39 | 10 | 84 | 0.007995 |
| rs2046210 (A) | JPT | G | 119 | 0,692 | A | 53 | 0,308 | 44 | 31 | 11 | 86 | 0.0003403 |
| rs2046210 (A) | CHD | G | 111 | 0,653 | A | 59 | 0,347 | 38 | 35 | 12 | 85 | 0.005972 |
| rs2046210 (A) | GIH | G | 120 | 0,682 | A | 56 | 0,318 | 42 | 36 | 10 | 88 | 0.0006855 |
| rs2046210 (A) | MEX | G | 79 | 0,79 | A | 21 | 0,21 | 32 | 15 | 3 | 50 | 3.175e06 |
| rs2046210 (A) | ASW | G | 42 | 0,404 | A | 62 | 0,596 | 7 | 28 | 17 | 52 | 0.07052 |
| rs2046210 (A) | MKK | G | 107 | 0,374 | A | 179 | 0,626 | 13 | 81 | 49 | 143 | 0.001143 |
| rs2046210 (A) | LWK | G | 71 | 0,394 | A | 109 | 0,606 | 15 | 41 | 34 | 90 | 0.01591 |
| rs2046210 (A) | YRI | G | 69 | 0,305 | A | 157 | 0,695 | 10 | 49 | 54 | 113 | 3.878e06 |
| rs2046210 (A) | TUN | G | 139 | 0,55 | A | 131 | 0,45 | 35 | 69 | 31 | 135 |  |
| rs720475 (A) | CEU | G | 165 | 0,737 | A | 59 | 0,263 | 59 | 47 | 6 | 112 | 1 |
| rs720475 (A) | TSI | G | 129 | 0,733 | A | 47 | 0,267 | 45 | 39 | 4 | 88 | 1 |
| rs720475 (A) | CHB | G | 159 | 0,946 | A | 9 | 0,054 | 75 | 9 | 0 | 84 | 7.131e08 |
| rs720475 (A) | JPT | G | 171 | 0,994 | A | 1 | 0,006 | 85 | 1 | 0 | 86 | 2.454e12 |
| rs720475 (A) | CHD | G | 165 | 0,971 | A | 5 | 0,029 | 80 | 5 | 0 | 85 | 6.374e10 |
| rs720475 (A) | GIH | G | 145 | 0,824 | A | 31 | 0,176 | 61 | 23 | 4 | 88 | 0.04353 |
| rs720475 (A) | MEX | G | 88 | 0,88 | A | 12 | 0,12 | 38 | 12 | 0 | 50 | 0.005314 |
| rs720475 (A) | ASW | G | 90 | 0,849 | A | 16 | 0,151 | 38 | 14 | 1 | 53 | 0.02914 |
| rs720475 (A) | MKK | G | 243 | 0,842 | A | 43 | 0,158 | 104 | 35 | 4 | 143 | 0.001462 |
| rs720475 (A) | LWK | G | 162 | 0,9 | A | 18 | 0,1 | 73 | 16 | 1 | 90 | 3.613e05 |
| rs720475 (A) | YRI | G | 213 | 0,942 | A | 13 | 0,058 | 100 | 13 | 0 | 113 | 2.597e09 |
| rs720475 (A) | TUN | G | 199 | 0,737 | A | 71 | 0,263 | 71 | 57 | 7 | 135 |  |
| rs10759243 (A) | CEU | C | 159 | 0,704 | A | 67 | 0,296 | 57 | 45 | 11 | 113 | 5.784e05 |
| rs10759243 (A) | TSI | C | 128 | 0,727 | A | 48 | 0,273 | 50 | 28 | 10 | 88 | 2.367e05 |
| rs10759243 (A) | CHB | C | 92 | 0,548 | A | 76 | 0,452 | 28 | 36 | 20 | 84 | 0.6749 |
| rs10759243 (A) | JPT | C | 98 | 0,57 | A | 74 | 0,43 | 26 | 46 | 14 | 86 | 0.3788 |
| rs10759243 (A) | CHD | C | 94 | 0,553 | A | 76 | 0,447 | 30 | 34 | 21 | 85 | 0.5955 |
| rs10759243 (A) | GIH | C | 116 | 0,659 | A | 60 | 0,341 | 41 | 34 | 13 | 88 | 0.005763 |
| rs10759243 (A) | MEX | C | 63 | 0,63 | A | 37 | 0,37 | 20 | 23 | 7 | 50 | 0.08301 |
| rs10759243 (A) | ASW | C | 53 | 0,5 | A | 53 | 0,5 | 13 | 27 | 13 | 53 | 0.7846 |
| rs10759243 (A) | MKK | C | 84 | 0,294 | A | 202 | 0,706 | 13 | 58 | 72 | 143 | 6.653e08 |
| rs10759243 (A) | LWK | C | 42 | 0,233 | A | 138 | 0,767 | 3 | 36 | 51 | 90 | 1.811e09 |
| rs10759243 (A) | YRI | C | 71 | 0,314 | A | 155 | 0,686 | 12 | 47 | 54 | 113 | 4.79e06 |
| rs10759243 (A) | TUN | C | 141 | 0,523 | A | 129 | 0,477 | 35 | 71 | 29 | 135 |  |
| rs7072776 (A) | CEU | G | 165 | 0,73 | A | 61 | 0,27 | 61 | 43 | 9 | 113 | 0.0001197 |
| rs7072776 (A) | TSI | G | 123 | 0,699 | A | 53 | 0,301 | 44 | 35 | 9 | 88 | 0.004223 |
| rs7072776 (A) | CHB | G | 162 | 0,964 | A | 6 | 0,036 | 78 | 6 | 0 | 84 | 2.2e16 |
| rs7072776 (A) | JPT | G | 90 | 1 | A | 0 | 0 | 45 | 0 | 0 | 45 | 3.783e14 |
| rs7072776 (A) | CHD | G | 160 | 0,952 | A | 8 | 0,048 | 76 | 8 | 0 | 84 | 2.2e16 |
| rs7072776 (A) | GIH | G | 132 | 0,75 | A | 44 | 0,25 | 52 | 28 | 8 | 88 | 6.666e05 |
| rs7072776 (A) | MEX | G | 65 | 0,65 | A | 35 | 0,35 | 20 | 25 | 5 | 50 | 0.146 |
| rs7072776 (A) | ASW | G | 58 | 0,547 | A | 48 | 0,453 | 14 | 30 | 9 | 53 | 0.9228 |
| rs7072776 (A) | MKK | G | 143 | 0,5 | A | 143 | 0,5 | 38 | 67 | 38 | 143 | 0.1888 |
| rs7072776 (A) | LWK | G | 66 | 0,371 | A | 112 | 0,629 | 14 | 38 | 37 | 89 | 0.0001392 |
| rs7072776 (A) | YRI | G | 86 | 0,381 | A | 140 | 0,619 | 14 | 58 | 41 | 113 | 0.0001051 |
| rs7072776 (A) | TUN | G | 151 | 0,559 | A | 119 | 0,441 | 39 | 73 | 23 | 135 |  |
| rs2380205 (T) | CEU | C | 116 | 0,518 | T | 108 | 0,482 | 30 | 56 | 26 | 112 | 0.9297 |
| rs2380205 (T) | TSI | C | 97 | 0,551 | T | 79 | 0,449 | 27 | 43 | 18 | 88 | 0.6711 |
| rs2380205 (T) | CHB | C | 152 | 0,905 | T | 16 | 0,095 | 70 | 12 | 2 | 84 | 5.4e16 |
| rs2380205 (T) | JPT | C | 156 | 0,907 | T | 16 | 0,093 | 70 | 16 | 0 | 86 | 2.2e16 |
| rs2380205 (T) | CHD | C | 148 | 0,871 | T | 22 | 0,129 | 66 | 16 | 3 | 85 | 2.422e13 |
| rs2380205 (T) | GIH | C | 134 | 0,77 | T | 40 | 0,23 | 50 | 34 | 3 | 87 | 3.794e07 |
| rs2380205 (T) | MEX | C | 78 | 0,78 | T | 22 | 0,22 | 33 | 12 | 5 | 50 | 1.697e05 |
| rs2380205 (T) | ASW | C | 43 | 0,406 | T | 63 | 0,594 | 10 | 23 | 20 | 53 | 0.04724 |
| rs2380205 (T) | MKK | C | 105 | 0,367 | T | 181 | 0,633 | 17 | 71 | 55 | 143 | 0.0002325 |
| rs2380205 (T) | LWK | C | 75 | 0,426 | T | 101 | 0,574 | 20 | 35 | 33 | 88 | 0.04954 |
| rs2380205 (T) | YRI | C | 89 | 0,394 | T | 137 | 0,606 | 18 | 53 | 42 | 113 | 0.004408 |
| rs2380205 (T) | TUN | C | 142 | 0,52 | T | 128 | 0,48 | 34 | 74 | 27 | 135 |  |
| rs704010 (T) | CEU | C | 127 | 0,567 | T | 97 | 0,433 | 36 | 55 | 21 | 112 | 0.02916 |
| rs704010 (T) | TSI | C | 105 | 0,597 | T | 71 | 0,403 | 31 | 43 | 14 | 88 | 0.16 |
| rs704010 (T) | CHB | C | 124 | 0,738 | T | 44 | 0,262 | 45 | 34 | 5 | 84 | 0.1413 |
| rs704010 (T) | JPT | C | 138 | 0,802 | T | 34 | 0,198 | 56 | 26 | 4 | 86 | 0.002821 |
| rs704010 (T) | CHD | C | 107 | 0,629 | T | 63 | 0,371 | 34 | 39 | 12 | 85 | 0.4863 |
| rs704010 (T) | GIH | C | 132 | 0,75 | T | 44 | 0,25 | 49 | 34 | 5 | 88 | 0.07663 |
| rs704010 (T) | MEX | C | 59 | 0,59 | T | 41 | 0,41 | 18 | 23 | 9 | 50 | 0.2124 |
| rs704010 (T) | ASW | C | 99 | 0,934 | T | 7 | 0,066 | 46 | 7 | 0 | 53 | 2.003e07 |
| rs704010 (T) | MKK | C | 245 | 0,857 | T | 41 | 0,143 | 105 | 35 | 3 | 143 | 2.271e07 |
| rs704010 (T) | LWK | C | 177 | 0,983 | T | 3 | 0,017 | 87 | 3 | 0 | 90 | 1.161e15 |
| rs704010 (T) | YRI | C | 222 | 0,982 | T | 4 | 0,018 | 109 | 4 | 0 | 113 | 2.2e16 |
| rs704010 (T) | TUN | C | 180 | 0,678 | T | 90 | 0,322 | 59 | 62 | 14 | 135 |  |
| rs1219648 (G) | CEU | A | 121 | 0,535 | G | 105 | 0,465 | 37 | 47 | 29 | 113 | 1 |
| rs1219648 (G) | TSI | A | 106 | 0,602 | G | 70 | 0,398 | 34 | 38 | 16 | 88 | 0.1815 |
| rs1219648 (G) | CHB | A | 103 | 0,613 | G | 65 | 0,387 | 29 | 45 | 10 | 84 | 0.1241 |
| rs1219648 (G) | JPT | A | 117 | 0,688 | G | 53 | 0,312 | 39 | 39 | 7 | 85 | 0.001802 |
| rs1219648 (G) | CHD | A | 113 | 0,673 | G | 55 | 0,327 | 35 | 43 | 6 | 84 | 0.005456 |
| rs1219648 (G) | GIH | A | 104 | 0,591 | G | 72 | 0,409 | 28 | 48 | 12 | 88 | 0.2719 |
| rs1219648 (G) | MEX | A | 54 | 1 | G | 46 | 0 | 16 | 22 | 12 | 50 | 1 |
| rs1219648 (G) | ASW | A | 59 | 0,567 | G | 45 | 0,433 | 16 | 27 | 9 | 52 | 0.6347 |
| rs1219648 (G) | MKK | A | 171 | 0,598 | G | 115 | 0,402 | 55 | 61 | 27 | 143 | 0.1471 |
| rs1219648 (G) | LWK | A | 124 | 0,689 | G | 56 | 0,311 | 40 | 44 | 6 | 90 | 0.001394 |
| rs1219648 (G) | YRI | A | 120 | 0,536 | G | 104 | 0,464 | 31 | 58 | 23 | 112 | 1 |
| rs1219648 (G) | TUN | A | 144 | 0,534 | G | 126 | 0,466 | 43 | 58 | 34 | 135 |  |
| rs2981582 (A) | CEU | G | 123 | 0,544 | A | 103 | 0,456 | 38 | 47 | 28 | 113 | 0.9442 |
| rs2981582 (A) | TSI | G | 106 | 0,544 | A | 70 | 0,456 | 34 | 38 | 16 | 88 | 0.2077 |
| rs2981582 (A) | CHB | G | 113 | 0,544 | A | 55 | 0,456 | 37 | 39 | 8 | 84 | 0.006841 |
| rs2981582 (A) | JPT | G | 132 | 0,544 | A | 40 | 0,456 | 50 | 32 | 4 | 86 | 1.736e06 |
| rs2981582 (A) | CHD | G | 121 | 0,544 | A | 49 | 0,456 | 41 | 39 | 5 | 85 | 0.0003856 |
| rs2981582 (A) | GIH | G | 109 | 0,544 | A | 67 | 0,456 | 33 | 43 | 12 | 88 | 0.1058 |
| rs2981582 (A) | MEX | G | 53 | 0,544 | A | 47 | 0,456 | 15 | 23 | 12 | 50 | 0.9975 |
| rs2981582 (A) | ASW | G | 55 | 0,544 | A | 51 | 0,456 | 11 | 33 | 9 | 53 | 0.8393 |
| rs2981582 (A) | MKK | G | 140 | 0,544 | A | 146 | 0,456 | 32 | 76 | 35 | 143 | 0.3004 |
| rs2981582 (A) | LWK | G | 97 | 0,544 | A | 83 | 0,456 | 21 | 55 | 14 | 90 | 1 |
| rs2981582 (A) | YRI | G | 110 | 0,544 | A | 116 | 0,456 | 24 | 62 | 27 | 113 | 0.3047 |
| rs2981582 (A) | TUN | G | 145 | 0,537 | A | 125 | 0,463 | 42 | 61 | 32 | 135 |  |
| rs3903072 (T) | CEU | G | 125 | 0,573 | T | 93 | 0,427 | 28 | 69 | 12 | 109 | 0.2634 |
| rs3903072 (T) | TSI | G | 90 | 0,529 | T | 80 | 0,471 | 23 | 44 | 18 | 85 | 0.9007 |
| rs3903072 (T) | CHB | G | 139 | 0,837 | T | 27 | 0,163 | 56 | 27 | 0 | 83 | 3.323e11 |
| rs3903072 (T) | JPT | G | 124 | 0,747 | T | 42 | 0,253 | 47 | 30 | 6 | 83 | 3.506e06 |
| rs3903072 (T) | CHD | G | ND | ND | T | ND | ND | ND | ND | ND | ND |  |
| rs3903072 (T) | GIH | G | ND | ND | T | ND | ND | ND | ND | ND | ND |  |
| rs3903072 (T) | MEX | G | 71 | 0,71 | T | 29 | 0,29 | 27 | 17 | 6 | 50 | 0.001443 |
| rs3903072 (T) | ASW | G | 83 | 0,814 | T | 19 | 0,186 | 33 | 17 | 1 | 51 | 4.083e07 |
| rs3903072 (T) | MKK | G | ND | ND | T | ND | ND | ND | ND | ND | ND |  |
| rs3903072 (T) | LWK | G | ND | ND | T | ND | ND | ND | ND | ND | ND |  |
| rs3903072 (T) | YRI | G | 196 | 0,891 | T | 24 | 0,109 | 87 | 22 | 1 | 110 | 2.2e16 |
| rs3903072 (T) | TUN | G | 140 | 0,533 | T | 130 | 0,467 | 39 | 62 | 34 | 135 |  |
| rs3817198 (C) | CEU | T | 152 | 0,673 | C | 74 | 0,327 | 50 | 52 | 11 | 113 | 0.2321 |
| rs3817198 (C) | TSI | T | 117 | 0,665 | C | 59 | 0,335 | 37 | 43 | 8 | 88 | 0.2027 |
| rs3817198 (C) | CHB | T | 153 | 0,911 | C | 15 | 0,089 | 71 | 11 | 2 | 84 | 5.33e06 |
| rs3817198 (C) | JPT | T | 151 | 0,878 | C | 21 | 0,122 | 65 | 21 | 0 | 86 | 0.0002391 |
| rs3817198 (C) | CHD | T | 154 | 0,906 | C | 16 | 0,094 | 71 | 12 | 2 | 85 | 9.195e06 |
| rs3817198 (C) | GIH | T | 115 | 0,653 | C | 61 | 0,347 | 40 | 35 | 13 | 88 | 0.1275 |
| rs3817198 (C) | MEX | T | 78 | 0,78 | C | 22 | 0,22 | 31 | 16 | 3 | 50 | 0.3574 |
| rs3817198 (C) | ASW | T | 98 | 0,925 | C | 8 | 0,075 | 45 | 8 | 0 | 53 | 4.967e05 |
| rs3817198 (C) | MKK | T | 235 | 0,822 | C | 51 | 0,178 | 96 | 43 | 4 | 143 | 0.009283 |
| rs3817198 (C) | LWK | T | 137 | 0,761 | C | 43 | 0,239 | 50 | 37 | 3 | 90 | 0.4691 |
| rs3817198 (C) | YRI | T | 204 | 0,903 | C | 22 | 0,097 | 91 | 22 | 0 | 113 | 1.25e06 |
| rs3817198 (C) | TUN | T | 196 | 0,723 | C | 74 | 0,277 | 72 | 52 | 11 | 135 |  |
| rs1292011 (G) | CEU | A | 137 | 0,606 | G | 89 | 0,394 | 40 | 57 | 16 | 113 | 0.1689 |
| rs1292011 (G) | TSI | A | 103 | 0,585 | G | 73 | 0,415 | 26 | 51 | 11 | 88 | 0.4081 |
| rs1292011 (G) | CHB | A | 128 | 0,762 | G | 40 | 0,238 | 51 | 26 | 7 | 84 | 5.393e06 |
| rs1292011 (G) | JPT | A | 133 | 0,773 | G | 39 | 0,227 | 52 | 29 | 5 | 86 | 1.307e06 |
| rs1292011 (G) | CHD | A | 139 | 0,827 | G | 29 | 0,173 | 55 | 29 | 0 | 84 | 1.794e09 |
| rs1292011 (G) | GIH | A | 65 | 0,369 | G | 111 | 0,631 | 8 | 49 | 31 | 88 | 0.0005668 |
| rs1292011 (G) | MEX | A | 62 | 0,62 | G | 38 | 0,38 | 17 | 28 | 5 | 50 | 0.2125 |
| rs1292011 (G) | ASW | A | 63 | 0,594 | G | 43 | 0,406 | 19 | 25 | 9 | 53 | 0.4089 |
| rs1292011 (G) | MKK | A | 175 | 0,616 | G | 109 | 0,384 | 58 | 59 | 25 | 142 | 0.08686 |
| rs1292011 (G) | LWK | A | 89 | 0,5 | G | 89 | 0,5 | 25 | 39 | 25 | 89 | 0.4543 |
| rs1292011 (G) | YRI | A | 134 | 0,593 | G | 92 | 0,407 | 37 | 60 | 16 | 113 | 0.2818 |
| rs1292011 (G) | TUN | A | 146 | 0,531 | G | 124 | 0,469 | 39 | 68 | 28 | 135 |  |
| rs2588809 (T) | CEU | C | 183 | 0,817 | T | 41 | 0,183 | 73 | 37 | 2 | 112 | 0.001189 |
| rs2588809 (T) | TSI | C | 131 | 0,753 | T | 43 | 0,247 | 48 | 35 | 4 | 87 | 0.1528 |
| rs2588809 (T) | CHB | C | 163 | 0,97 | T | 5 | 0,03 | 79 | 5 |  | 84 | 1.692e12 |
| rs2588809 (T) | JPT | C | 165 | 0,959 | T | 7 | 0,041 | 80 | 5 | 1 | 86 | 1.04e11 |
| rs2588809 (T) | CHD | C | 165 | 0,994 | T | 1 | 0,006 | 82 | 1 |  | 83 | 9.634e15 |
| rs2588809 (T) | GIH | C | 133 | 0,782 | T | 37 | 0,218 | 49 | 35 | 1 | 85 | 0.03506 |
| rs2588809 (T) | MEX | C | 85 | 0,85 | T | 15 | 0,15 | 35 | 15 |  | 50 | 0.002378 |
| rs2588809 (T) | ASW | C | 76 | 0,717 | T | 30 | 0,283 | 27 | 22 | 4 | 53 | 0.6329 |
| rs2588809 (T) | MKK |  | 88 | 0,634 |  | 18 | 0,366 | 44 |  | 9 | 53 | 0.006766 |
| rs2588809 (T) | LWK | C | 141 | 0,694 | T | 65 | 0,306 | 52 | 37 | 14 | 103 | 1 |
| rs2588809 (T) | YRI | C | 198 | 0,735 | T | 92 | 0,265 | 61 | 76 | 8 | 145 | 1 |
| rs2588809 (T) | TUN | C | 185 | 0,702 | T | 85 | 0,298 | 67 | 51 | 17 | 135 |  |
| rs941764 (G) | CEU | A | 148 | 0,661 | G | 76 | 0,339 | 50 | 48 | 14 | 112 | 0.002542 |
| rs941764 (G) | TSI | A | 120 | 0,682 | G | 56 | 0,318 | 41 | 38 | 9 | 88 | 0.001173 |
| rs941764 (G) | CHB | A | 139 | 0,827 | G | 29 | 0,173 | 58 | 23 | 3 | 84 | 1.959e10 |
| rs941764 (G) | JPT | A | 154 | 0,895 | G | 18 | 0,105 | 69 | 16 | 1 | 86 | 1.107e15 |
| rs941764 (G) | CHD | A | 154 | 0,906 | G | 16 | 0,094 | 70 | 14 | 1 | 85 | 2.2e16 |
| rs941764 (G) | GIH | A | 135 | 0,776 | G | 39 | 0,224 | 51 | 33 | 3 | 87 | 1.296e07 |
| rs941764 (G) | MEX | A | 55 | 0,55 | G | 45 | 0,45 | 17 | 21 | 12 | 50 | 0.7202 |
| rs941764 (G) | ASW | A | 29 | 0,274 | G | 77 | 0,726 | 5 | 19 | 29 | 53 | 2.202e05 |
| rs941764 (G) | MKK | A | 77 | 0,269 | G | 209 | 0,731 | 8 | 61 | 74 | 143 | 1.744e09 |
| rs941764 (G) | LWK | A | 44 | 0,244 | G | 136 | 0,756 | 4 | 36 | 50 | 90 | 7.966e09 |
| rs941764 (G) | YRI | A | 40 | 0,177 | G | 186 | 0,823 | 3 | 34 | 76 | 113 | 3.827e15 |
| rs941764 (G) | TUN | A | 141 | 0,532 | G | 129 | 0,468 | 37 | 67 | 31 | 135 |  |
| rs3803662 (A) | CEU | G | 170 | 0,752 | A | 56 | 0,248 | 65 | 40 | 8 | 113 | 3.428e05 |
| rs3803662 (A) | TSI | G | 122 | 0,693 | A | 54 | 0,307 | 40 | 42 | 6 | 88 | 0.01206 |
| rs3803662 (A) | CHB | G | 53 | 0,315 | A | 115 | 0,685 | 9 | 35 | 40 | 84 | 3.446e07 |
| rs3803662 (A) | JPT | G | 69 | 0,401 | A | 103 | 0,599 | 15 | 39 | 32 | 86 | 0.0007478 |
| rs3803662 (A) | CHD | G | 61 | 0,363 | A | 107 | 0,637 | 11 | 39 | 34 | 84 | 3.771e05 |
| rs3803662 (A) | GIH | G | 129 | 0,733 | A | 47 | 0,267 | 49 | 31 | 8 | 88 | 0.0007134 |
| rs3803662 (A) | MEX | G | 61 | 0,61 | A | 39 | 0,39 | 18 | 25 | 7 | 50 | 0.5704 |
| rs3803662 (A) | ASW | G | 51 | 0,481 | A | 55 | 0,519 | 11 | 29 | 13 | 53 | 0.1475 |
| rs3803662 (A) | MKK | G | 126 | 0,441 | A | 160 | 0,559 | 28 | 70 | 45 | 143 | 0.002931 |
| rs3803662 (A) | LWK | G | 78 | 0,433 | A | 102 | 0,567 | 19 | 40 | 31 | 90 | 0.005899 |
| rs3803662 (A) | YRI | G | 104 | 0,464 | A | 120 | 0,536 | 24 | 56 | 32 | 112 | 0.02385 |
| rs3803662 (A) | TUN | G | 154 | 0,586 | A | 116 | 0,414 | 45 | 64 | 26 | 135 |  |
| rs8051542 (T) | CEU | C | 124 | 0,549 | T | 102 | 0,451 | 35 | 54 | 24 | 113 | 0.6935 |
| rs8051542 (T) | TSI | C | 108 | 0,614 | T | 68 | 0,386 | 31 | 46 | 11 | 88 | 0.4186 |
| rs8051542 (T) | CHB | C | 141 | 0,839 | T | 27 | 0,161 | 58 | 25 | 1 | 84 | 9.975e09 |
| rs8051542 (T) | JPT | C | 134 | 0,628 | T | 38 | 0,372 | 50 | 34 | 2 | 86 | 1.147e05 |
| rs8051542 (T) | CHD | C | 136 | 0,8 | T | 34 | 0,2 | 57 | 22 | 6 | 85 | 1.269e06 |
| rs8051542 (T) | GIH | C | 122 | 0,693 | T | 54 | 0,307 | 41 | 40 | 7 | 88 | 0.01206 |
| rs8051542 (T) | MEX | C | 56 | 0,56 | T | 44 | 0,44 | 17 | 22 | 11 | 50 | 0.9516 |
| rs8051542 (T) | ASW | C | 72 | 0,679 | T | 34 | 0,321 | 26 | 20 | 7 | 53 | 0.06834 |
| rs8051542 (T) | MKK | C | 179 | 0,626 | T | 107 | 0,374 | 57 | 65 | 21 | 143 | 0.212 |
| rs8051542 (T) | LWK | C | 113 | 0,628 | T | 67 | 0,372 | 32 | 49 | 9 | 90 | 0.2642 |
| rs8051542 (T) | YRI | C | 174 | 0,77 | T | 52 | 0,23 | 69 | 36 | 8 | 113 | 4.623e06 |
| rs8051542 (T) | TUN | C | 154 | 0,604 | T | 116 | 0,396 | 45 | 64 | 26 | 135 |  |
| rs13329835 (G) | CEU | A | 185 | 0,819 | G | 41 | 0,181 | 74 | 37 | 2 | 113 | 7.408e07 |
| rs13329835 (G) | TSI | A | 127 | 0,722 | G | 49 | 0,278 | 45 | 37 | 6 | 88 | 0.02164 |
| rs13329835 (G) | CHB | A | 162 | 0,964 | G | 6 | 0,036 | 78 | 6 | 0 | 84 | 3.644e16 |
| rs13329835 (G) | JPT | A | 168 | 0,977 | G | 4 | 0,023 | 82 | 4 | 0 | 86 | 2.2e16 |
| rs13329835 (G) | CHD | A | 158 | 0,929 | G | 12 | 0,071 | 73 | 12 | 0 | 85 | 4.246e13 |
| rs13329835 (G) | GIH | A | 159 | 0,903 | G | 17 | 0,097 | 73 | 13 | 2 | 88 | 2.745e11 |
| rs13329835 (G) | MEX | A | 89 | 0,89 | G | 11 | 0,11 | 40 | 9 | 1 | 50 | 5.467e07 |
| rs13329835 (G) | ASW | A | 48 | 0,453 | G | 58 | 0,547 | 13 | 22 | 18 | 53 | 0.007564 |
| rs13329835 (G) | MKK | A | 98 | 0,348 | G | 184 | 0,652 | 16 | 66 | 59 | 141 | 9.764e10 |
| rs13329835 (G) | LWK | A | 52 | 0,289 | G | 128 | 0,711 | 9 | 34 | 47 | 90 | 3.969e11 |
| rs13329835 (G) | YRI | A | 52 | 0,23 | G | 174 | 0,77 | 5 | 42 | 66 | 113 | 2.2e16 |
| rs13329835 (G) | TUN | A | 165 | 0,624 | G | 105 | 0,376 | 50 | 65 | 20 | 135 |  |
| rs9911630 (G) | CEU | A | 150 | 0,664 | G | 76 | 0,336 | 49 | 52 | 12 | 113 | 0.06175 |
| rs9911630 (G) | TSI | A | 107 | 0,608 | G | 69 | 0,392 | 31 | 45 | 12 | 88 | 0.5928 |
| rs9911630 (G) | CHB | A | 113 | 0,673 | G | 55 | 0,327 | 39 | 35 | 10 | 84 | 0.05987 |
| rs9911630 (G) | JPT | A | 125 | 0,727 | G | 47 | 0,273 | 45 | 35 | 6 | 86 | 0.002128 |
| rs9911630 (G) | CHD | A | 95 | 0,565 | G | 73 | 0,435 | 23 | 49 | 12 | 84 | 0.8778 |
| rs9911630 (G) | GIH | A | 92 | 0,523 | G | 84 | 0,477 | 23 | 46 | 19 | 88 | 0.2955 |
| rs9911630 (G) | MEX | A | 75 | 0,75 | G | 25 | 0,25 | 29 | 17 | 4 | 50 | 0.003535 |
| rs9911630 (G) | ASW | A | 40 | 0,377 | G | 66 | 0,623 | 6 | 28 | 19 | 53 | 0.0007103 |
| rs9911630 (G) | MKK | A | 133 | 0,465 | G | 153 | 0,535 | 30 | 73 | 40 | 143 | 0.01004 |
| rs9911630 (G) | LWK | A | 49 | 0,272 | G | 131 | 0,728 | 9 | 31 | 50 | 90 | 3.397e10 |
| rs9911630 (G) | YRI | A | 46 | 0,205 | G | 178 | 0,795 | 7 | 32 | 73 | 112 | 2.2e16 |
| rs9911630 (G) | TUN | A | 156 | 0,574 | G | 114 | 0,426 | 44 | 68 | 23 | 135 |  |
| rs799916 (T) | CEU | G | 76 | 0,336 | T | 150 | 0,664 | 12 | 52 | 49 | 113 | 3.37e08 |
| rs799916 (T) | TSI | G | 67 | 0,385 | T | 107 | 0,615 | 11 | 45 | 31 | 87 | 4.188e05 |
| rs799916 (T) | CHB | G | 55 | 0,331 | T | 111 | 0,669 | 10 | 35 | 38 | 83 | 2.974e07 |
| rs799916 (T) | JPT | G | 47 | 0,273 | T | 125 | 0,727 | 6 | 35 | 45 | 86 | 1.683e10 |
| rs799916 (T) | CHD | G | 72 | 0,429 | T | 96 | 0,571 | 12 | 48 | 24 | 84 | 0.001527 |
| rs799916 (T) | GIH | G | 84 | 0,477 | T | 92 | 0,523 | 19 | 46 | 23 | 88 | 0.02667 |
| rs799916 (T) | MEX | G | 38 | 0,388 | T | 60 | 0,612 | 9 | 20 | 20 | 49 | 0.0009622 |
| rs799916 (T) | ASW | G | 65 | 0,613 | T | 41 | 0,387 | 18 | 29 | 6 | 53 | 0.7523 |
| rs799916 (T) | MKK | G | 153 | 0,535 | T | 133 | 0,465 | 40 | 73 | 30 | 143 | 0.232 |
| rs799916 (T) | LWK | G | 129 | 0,717 | T | 51 | 0,283 | 48 | 33 | 9 | 90 | 0.007671 |
| rs799916 (T) | YRI | G | 175 | 0,774 | T | 51 | 0,226 | 69 | 37 | 7 | 113 | 1.788e05 |
| rs799916 (T) | TUN | G | 159 | 0,596 | T | 111 | 0,404 | 44 | 71 | 20 | 135 |  |
| rs1436904 (G) | CEU | T | 128 | 0,566 | G | 98 | 0,434 | 38 | 52 | 23 | 113 | 0.01382 |
| rs1436904 (G) | TSI | T | 95 | 0,546 | G | 79 | 0,454 | 22 | 51 | 14 | 87 | 0.006899 |
| rs1436904 (G) | CHB | T | 75 | 0,452 | G | 91 | 0,548 | 12 | 51 | 20 | 83 | 5.091e06 |
| rs1436904 (G) | JPT | T | 99 | 0,576 | G | 73 | 0,424 | 29 | 41 | 16 | 86 | 0.03769 |
| rs1436904 (G) | CHD | T | 107 | 0,637 | G | 61 | 0,363 | 36 | 35 | 13 | 84 | 0.438 |
| rs1436904 (G) | GIH | T | 144 | 0,818 | G | 32 | 0,182 | 60 | 24 | 4 | 88 | 0.001539 |
| rs1436904 (G) | MEX | T | 58 | 0,58 | G | 42 | 0,42 | 15 | 28 | 7 | 50 | 0.1031 |
| rs1436904 (G) | ASW | T | 75 | 0,708 | G | 31 | 0,292 | 25 | 25 | 3 | 53 | 0.6627 |
| rs1436904 (G) | MKK | T | 200 | 0,699 | G | 86 | 0,301 | 66 | 68 | 9 | 143 | 0.6482 |
| rs1436904 (G) | LWK | T | 134 | 0,744 | G | 46 | 0,256 | 48 | 38 | 4 | 90 | 0.1577 |
| rs1436904 (G) | YRI | T | 183 | 0,81 | G | 43 | 0,19 | 73 | 37 | 3 | 113 | 0.001257 |
| rs1436904 (G) | TUN | T | 183 | 0,708 | G | 87 | 0,292 | 63 | 57 | 15 | 135 |  |
| rs4808801 (G) | CEU | A | 148 | 0,655 | G | 78 | 0,345 | 53 | 42 | 18 | 113 | 0.1832 |
| rs4808801 (G) | TSI | A | 121 | 0,688 | G | 55 | 0,312 | 42 | 37 | 9 | 88 | 0.05376 |
| rs4808801 (G) | CHB | A | 122 | 0,726 | G | 46 | 0,274 | 47 | 28 | 9 | 84 | 0.006209 |
| rs4808801 (G) | JPT | A | 129 | 0,75 | G | 43 | 0,25 | 50 | 29 | 7 | 86 | 0.001006 |
| rs4808801 (G) | CHD | A | 139 | 0,818 | G | 31 | 0,182 | 58 | 23 | 4 | 85 | 1.429e06 |
| rs4808801 (G) | GIH | A | 98 | 0,557 | G | 78 | 0,443 | 29 | 40 | 19 | 88 | 0.5159 |
| rs4808801 (G) | MEX | A | 58 | 0,58 | G | 42 | 0,42 | 14 | 30 | 6 | 50 | 0.9206 |
| rs4808801 (G) | ASW | A | 31 | 0,292 | G | 75 | 0,708 | 7 | 17 | 29 | 53 | 3.004e07 |
| rs4808801 (G) | MKK | A | 109 | 0,381 | G | 177 | 0,619 | 17 | 75 | 51 | 143 | 9.48e07 |
| rs4808801 (G) | LWK | A | 42 | 0,233 | G | 138 | 0,767 | 5 | 32 | 53 | 90 | 1.266e13 |
| rs4808801 (G) | YRI | A | 51 | 0,226 | G | 175 | 0,774 | 1 | 49 | 63 | 113 | 3.932e16 |
| rs4808801 (G) | TUN | A | 160 | 0,596 | G | 110 | 0,404 | 47 | 66 | 22 | 135 |  |
| rs6001930 (C) | CEU | T | 203 | 0,898 | C | 23 | 0,102 | 91 | 21 | 1 | 113 | 0.8497 |
| rs6001930 (C) | TSI | T | 162 | 0,92 | C | 14 | 0,08 | 74 | 14 | 0 | 88 | 0.3523 |
| rs6001930 (C) | CHB | T | 133 | 0,792 | C | 35 | 0,208 | 51 | 31 | 2 | 84 | 0.008172 |
| rs6001930 (C) | JPT | T | 112 | 0,651 | C | 60 | 0,349 | 38 | 36 | 12 | 86 | 3.028e09 |
| rs6001930 (C) | CHD | T | 143 | 0,841 | C | 27 | 0,159 | 60 | 23 | 2 | 85 | 0.1918 |
| rs6001930 (C) | GIH | T | 151 | 0,858 | C | 25 | 0,142 | 66 | 19 | 3 | 88 | 0.41 |
| rs6001930 (C) | MEX | T | 90 | 0,9 | C | 10 | 0,1 | 40 | 10 | 0 | 50 | 0.9067 |
| rs6001930 (C) | ASW | T | 92 | 0,868 | C | 14 | 0,132 | 39 | 14 | 0 | 53 | 0.696 |
| rs6001930 (C) | MKK | T | 251 | 0,878 | C | 35 | 0,122 | 110 | 31 | 2 | 143 | 0.7786 |
| rs6001930 (C) | LWK | T | 157 | 0,872 | C | 23 | 0,128 | 69 | 19 | 2 | 90 | 0.698 |
| rs6001930 (C) | YRI | T | 193 | 0,854 | C | 33 | 0,146 | 81 | 31 | 1 | 113 | 0.3043 |
| rs6001930 (C) | TUN | T | 240 | 0,891 | C | 30 | 0,109 | 107 | 26 | 2 | 135 |  |

**Table S4.** MicroRNA binding sites altered by the *BRCA1*-rs9911630 variant.

| **Accession** | **ID** | **Strand** | **Score** | **eValue** | **Loss or gain of the Micro RNAs binding sites** |
| --- | --- | --- | --- | --- | --- |
| MIMAT0015474 | bmo-miR-3287 | - | 61 | 7.7 | LOSS |
| MIMAT0032469 | ssa-miR-19d-5p | - | 60 | 9.3 | LOSS |
| MIMAT0024482 | mse-miR-2766 | - | 61 | 7.7 | GAIN |
