# Supplementary material for: A genome wide SNP genotyping study in the Tunisian population: specific reporting on a subset of common breast cancer risk loci
Source: BMC Cancer. 2018 Dec 29;18:1295. doi: 10.1186/s12885-018-5133-8 (PMC6310952; doi:10.1186/s12885-018-5133-8)

**Figure S1.**

**(a)**


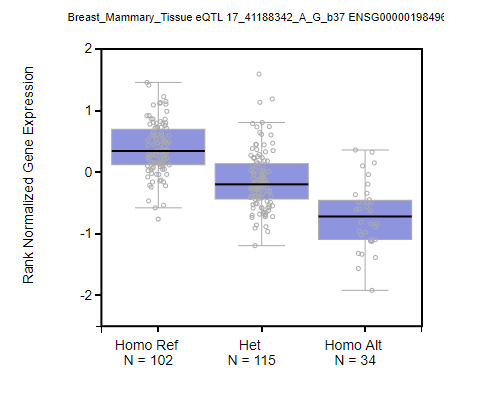


**(b)**


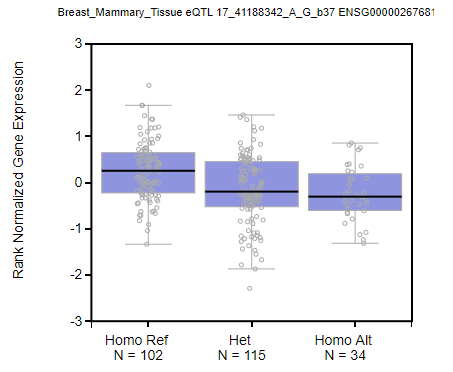


**(c)**


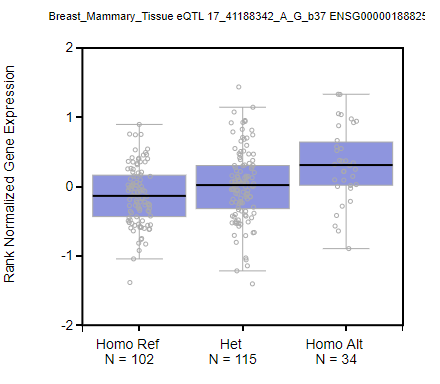


.

**Figure S2.**

**(a)** [**bmo-miR-3287**](http://www.mirbase.org/cgi-bin/mirna_entry.pl?acc=MIMAT0015474)

UserSeq ccacccuucaacac
 ||||||||||| ||
bmo-miR-3287 ccacccuucaaaac

**(b)** [**ssa-miR-19d-5p**](http://www.mirbase.org/cgi-bin/mirna_entry.pl?acc=MIMAT0032469)

UserSeq ggcugcccacccuucaacacu
 | |||||||||| ||| ||
ssa-miR-19d-5p gacugcccaccccgcaaagcu

**(c)** [**mse-miR-2766**](http://www.mirbase.org/cgi-bin/mirna_entry.pl?acc=MIMAT0024482)

UserSeq cccacccuucgaca
 |||||| |||||||
mse-miR-2766 cccaccauucgaca

.

**Figure S3.**

**(a)**


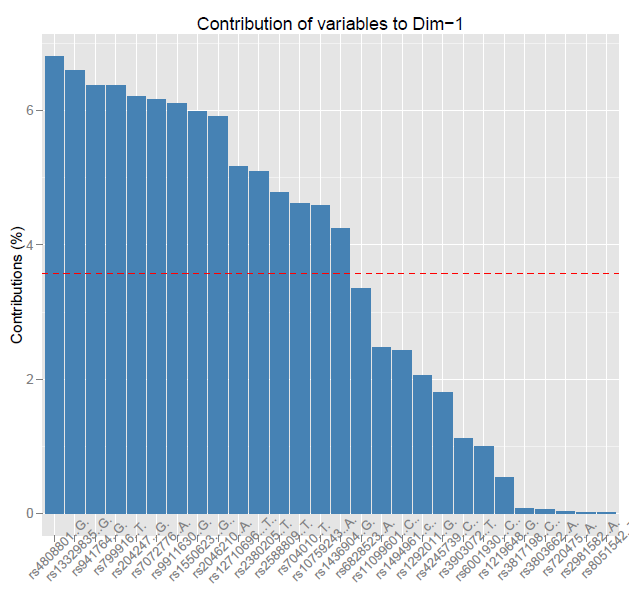


**(b)**


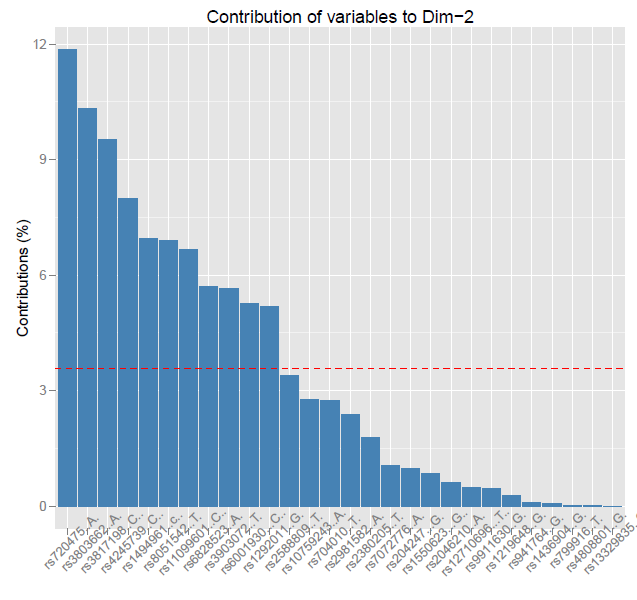


**Figure S4.**

**(a)**

**
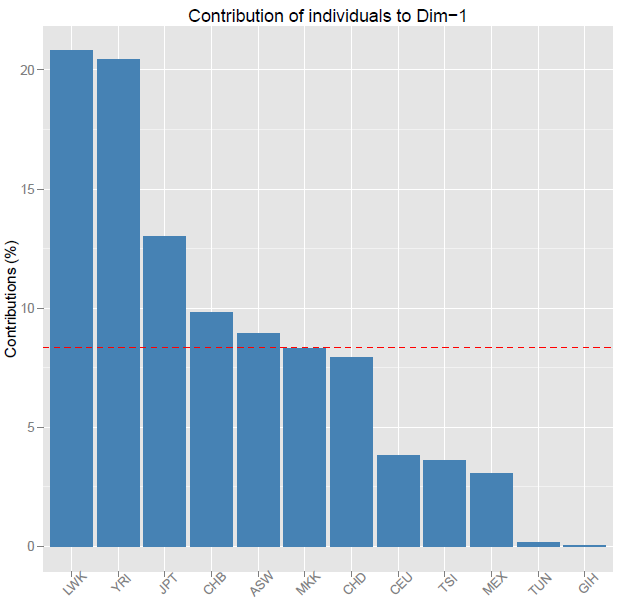
**

**(b)**

**
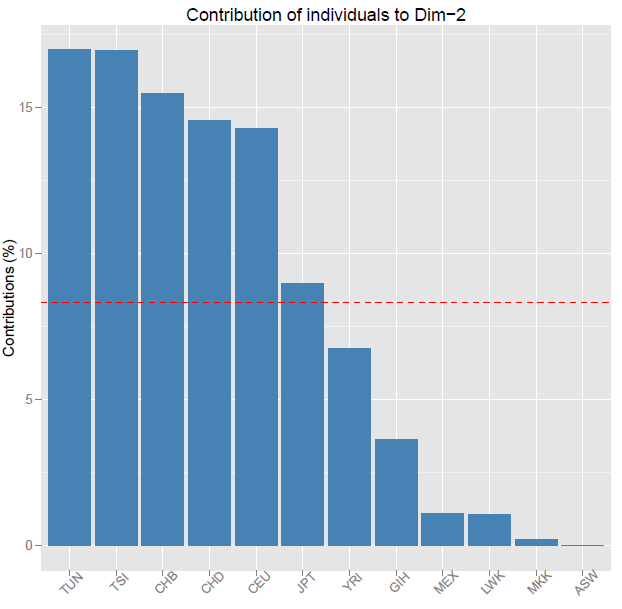
**

**Figure S5.**


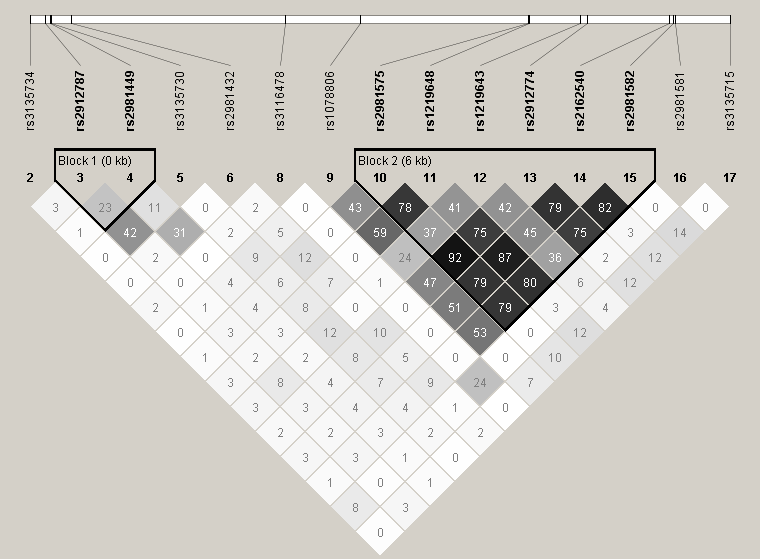

Supplement: Supplementary file 2 — Figure S1 GTEX Boxplots representing the most significant eQTL results for variant rs9911630 in breast mammary tissue. Box plots represent the expression levels of the indicated transcripts with respect to the rs9911630 genotypes. Expression levels are shown for (a) NBR2 gene, (b) CTD-3199 J23.6 gene and (c) LINC00910 gene. Horizontal bars indicate mean expression level per genotype. Additional information on the eQTL p values are reported in Table 1. Figure S2 Alignment of the sequence around rs9911630 with binding site of (a) bmo-miR-3287, (b) ssa-miR-19d-5p and (c) mse-miR-2766. The SNP is shown in red and the allele binding the microRNA is also shown. Figure S3 Contributions of variables (SNPs and populations) in Dim1 and Dim2. The contribution of each tested variable (based on their variants frequencies) in the general variability between the different selected populations shown on the PCA (a) to the first dimension of the PCA (Dim1 for the variability between African and non-African populations –see Fig. 3) and (b) to the second dimension of the PCA (Dim2 for the variability between European and non-European populations). Figure S4 Contributions of each tested population in the general variability between the different selected populations shown on the PCA (a) the first dimension of the PCA (Dim1) and (b) the second dimension of the PCA (Dim2). Figure S5 A map of the linkage disequilibrium in intron 2 of FGFR2 gene containing two SNPs associated with breast cancer risk in the Tunisian population (rs1219648 and rs2981582). (DOCX 263 kb) [file 12885_2018_5133_MOESM2_ESM.docx]
